# Physiological and molecular aspects of seed longevity: exploring intra‐species variation in eight *Pisum sativum* L. accessions

**DOI:** 10.1111/ppl.13698

**Published:** 2022-05-18

**Authors:** Maraeva Gianella, Enrico Doria, Daniele Dondi, Chiara Milanese, Lucia Gallotti, Andreas Börner, Lorena Zannino, Anca Macovei, Andrea Pagano, Filippo Guzzon, Marco Biggiogera, Alma Balestrazzi

**Affiliations:** ^1^ Department of Biology and Biotechnology ‘L. Spallanzani’ University of Pavia Pavia Italy; ^2^ Royal Botanic Gardens, Kew, Wakehurst, Ardingly Haywards Heath West Sussex UK; ^3^ C.S.G.I. & Department of Chemistry University of Pavia Pavia Italy; ^4^ Genebank Department Leibniz Institute of Plant Genetics and Crop Plant Research (IPK) Corrensstr Seeland Germany; ^5^ International Maize and Wheat Improvement Center (CIMMYT) Carretera México‐Veracruz Texcoco Mexico State Mexico; ^6^ Centre for Pacific Crops and Trees (CePaCT), Land Resource Division (LRD) Pacific Community (SPC) Suva Fiji

## Abstract

Conservation of plant genetic diversity is fundamental for crop improvement, increasing agricultural production and sustainability, especially in the face of climatic changes. Although seed longevity is essential for the management of seed banks, few studies have, so far, addressed differences in this trait among the accessions of a single species. Eight *Pisum sativum* L. (pea) accessions were investigated to study the impact of long‐term (approximately 20 years) storage, aiming to reveal contrasting seed longevity and clarify the causes for these differences. The outstanding seed longevity observed in the G4 accession provided a unique experimental system. To characterize the biochemical and physical status of stored seeds, reactive oxygen species, lipid peroxidation, tocopherols, free proline and reducing sugars were measured. Thermoanalytical measurements (thermogravimetry and differential scanning calorimetry) and transmission electron microscopy combined with immunohistochemical analysis were performed. The long‐lived G4 seeds neither consumed tocopherols during storage nor showed free proline accumulation, as a deterioration hallmark, whereas reducing sugars were not affected. Thermal decomposition suggested a biomass composition compatible with the presence of low molecular weight molecules. Expansion of heterochromatic areas and reduced occurrence of γH2AX foci were highlighted in the nucleus of G4 seeds. The longevity of G4 seeds correlates with the occurrence of a reducing cellular environment and a nuclear ultrastructure favourable to genome stability. This work brings novelty to the study of within‐species variations in seed longevity, underlining the relevance of multidisciplinary approaches in seed longevity research.

## 1 INTRODUCTION

*Ex situ *seed conservation is of key importance for preserving endangered plant species (Mondoni et al., 2011), conserving germplasm for forestry and restoration projects (Bonner, 1990; Merrit & Dixon, 2011) as well as in safeguarding the agronomic, biological, and cultural diversity connected with crop genetic resources (McLean‐Rodríguez et al., 2019; Veteläinen et al., 2009). Seed banking allows the long‐term conservation of orthodox seeds (desiccation and freezing tolerant seeds) with limited costs (Davies & Allender, 2017; Li & Pritchard, 2009; McCouch et al., 2013; Rivière & Müller, 2018). Seed banking is increasingly important considering the growing effects of climatic changes on plant diversity (Thuiller et al., 2005) and the need to enhance the accessibility and use of genetic diversity in crop improvement to increase agricultural production and sustainability in the face of global climate changes (Esquinas‐Alcázar, 2005; McCouch et al., 2013).

Despite the fundamental relevance of seed longevity for the management of seed banks (Colville & Pritchard, 2019), there are few available studies addressing the differences in seed longevity among accessions of a single plant species (Guzzon et al., 2021). Furthermore, the physiological and molecular bases of such a complex trait, shaped by genetic and environmental factors, still need to be fully clarified (Lee et al., 2019; Sano et al., 2015; Zinsmeister et al., 2020). Temperature and moisture content are the major exogenous factors influencing seed longevity in storage (Dickie et al., 1990; Ellis & Roberts, 1980; Roberts, 1973). Oxidative stress, the main driving force of seed aging, results from reactive oxygen species (ROS) accumulation and decreased antioxidant capacity (Kurek et al., 2019). Seed deterioration can be monitored by measuring the by‐products of lipid peroxidation (Bailly et al., 1996; Wiebach et al., 2020). Among the cellular antioxidants, tocopherols prevent membrane deterioration by scavenging lipid peroxy radicals during seed storage, and modifications in their biosynthesis result in reduced longevity (Chen et al., 2016). Oligosaccharides, favouring the seed glassy state by increasing cytoplasmic viscosity, can also improve storability (Buitink et al., 2000; Ebone et al., 2019; Lehner et al., 2008). However, according to Gurusinghe and Bradford (2001), changes in oligosaccharide contents alone cannot account for poor seed longevity, particularly in the context of post‐priming storage, whereas a negative correlation between monosaccharide levels and desiccation tolerance has been reported (Hoekstra et al., 2001).

Seed longevity is tightly linked to genome maintenance, as the loss of DNA integrity is a typical hallmark of seed deterioration (Cordoba‐Cañero et al., 2014; El‐Maarouf‐Bouteau et al., 2011; Kranner et al., 2010; López‐Fernández et al., 2018). Following long‐term storage, successful germination is dependent on the DNA damage response, namely a plethora of DNA damage sensing, signalling and repair pathways activated during imbibition (Balestrazzi et al., 2011; Diaz & Pecinka, 2017; Waterworth, Bray, & West, 2019; Waterworth et al., 2019). In deteriorated seeds, prolonged DNA repair is required before the onset of DNA replication (Elder et al., 1987). The cellular repair pathways can also influence seed longevity in response to environmental changes (Mondoni et al., 2014). In both animals and plants, phosphorylation of histone H2AX in the proximity of DNA double‐strand breaks (DSBs) sites is regarded as the earliest DNA damage response (DDR) hallmark which triggers DSB repair (Branzei & Foiani, 2008; Waterworth et al., 2011). The phosphorylated histone variant γH2AX reflects DNA damage accumulation and it has been used to assess DSB repair kinetics in actively dividing plant tissues (Charbonnel et al., 2010; Hirakawa & Matsunaga, 2019). According to Waterworth et al. (2019a), *Arabidopsis thaliana h2ax* mutants showed increased sensitivity to seed aging, highlighting the role of histone H2AX in promoting genome maintenance in the context of seed germination and longevity. Changes in chromatin structure also contribute to genome maintenance in the desiccated state as well as during rehydration (Waterworth et al.,  2019a; Waterworth et al., 2019b). High chromatin condensation protects against oxidative damage and double‐strand breakage (Falk et al., 2010; Takata et al., 2013). Increased chromatin compaction in seeds has been demonstrated to be part of the seed developmental programme associated with desiccation tolerance (van Zanten et al., 2011). On the other hand, reduced heterochromatin density has been reported in aged seeds as a consequence of DNA fragmentation (Begnami & Cortelazzo, 1996; Burrieza et al., 2016).

In the present work, eight *Pisum sativum* L. (pea) accessions were investigated using a multidisciplinary approach to dissect the impact of 20 years of storage on cold‐stored and room‐temperature stored seeds. Pea is a crop of major relevance worldwide, with an estimated global harvested area of 4.3 and 10.8% of the total area of vegetables and legumes production, respectively. Its production accounts for 2.7 and 30.6% of the global vegetables and legumes production (FAOSTAT, 2018). The long‐term conservation of pea genetic resources is fundamental for keeping available accessions that could serve as sources of favourable alleles (Holdsworth et al., 2017). The storage behaviour of pea seeds has been investigated in a few studies (Ballesteros & Walters, 2019; Ellis et al., 2018; Nagel & Börner, 2010; Redden & Partington, 2019), but differences in seed longevity observed among different genotypes still need to be fully clarified. A comparative, multidisciplinary analysis was performed in different pea accessions to define the contribution of different cellular and metabolic factors to their seed longevity profiles.

## 2 MATERIALS AND METHODS

### 2.1 Plant material and germination tests

**FIGURE 1:**
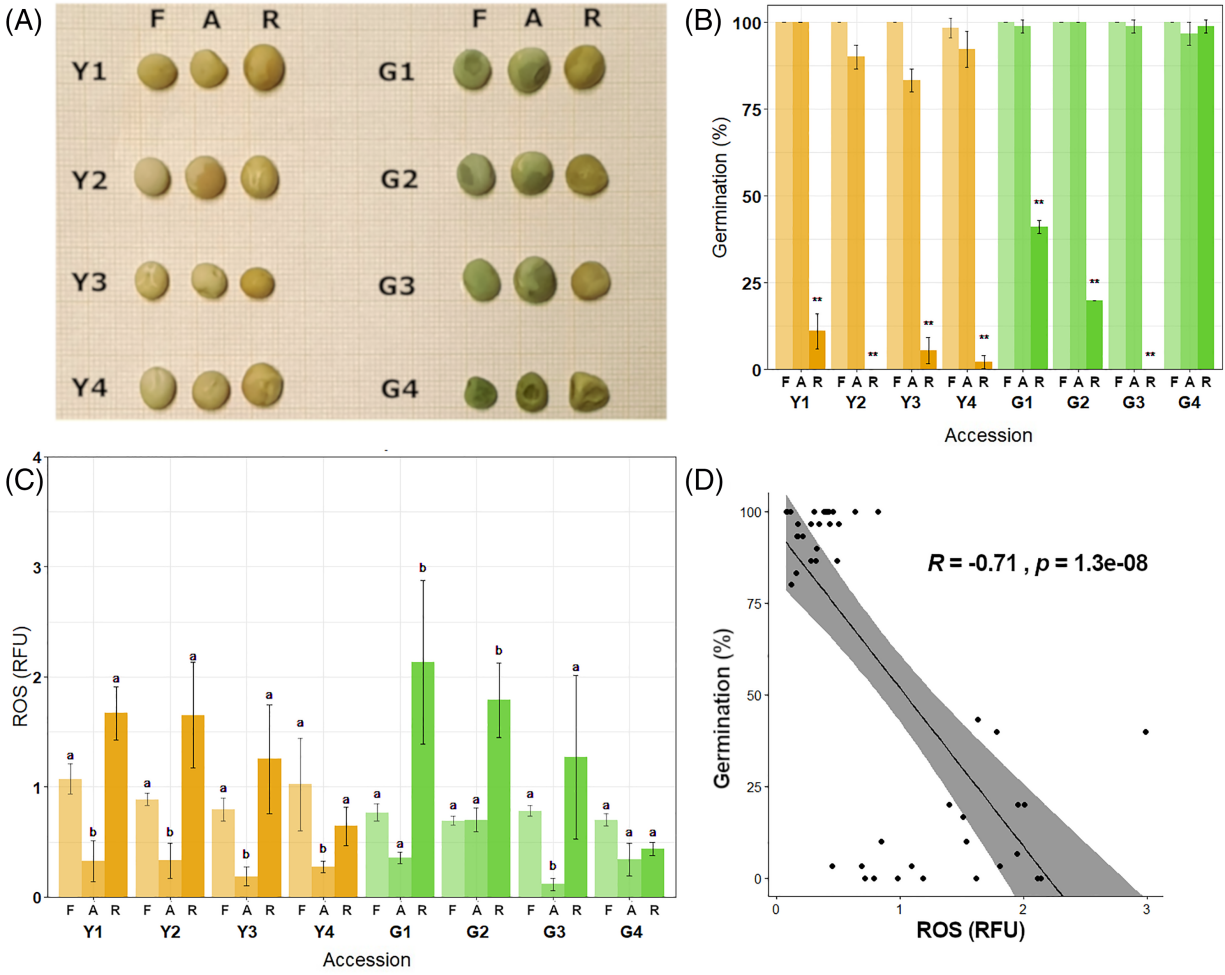
(A) Seed lots used in this study. Y, yellow. G, green. F, harvested in 2019 (fresh). A, harvested in 2001 and kept in cold storage (aged). R, harvested in 2001 and conserved at room temperature conditions. (B) Germination percentage of the 8 pea accessions (fresh seeds; seeds aged in cold storage and at room temperature conditions). Error bars represent the standard deviation of three biological replicates (20 seeds each). (C) ROS levels measured in dry pea seeds using the DCF‐DA fluorescent dye. Letters above bars represent statistically significant differences (GLM with Tukey post‐hoc test, *P* < 0.05) within the same accession. R.F.U., relative fluorescence unit. ROS, reactive oxygen species. DCF‐DA, dye 2′,7′‐dichlorofluorescein diacetate. Asterisks represent statistically significant differences between aged and fresh seeds within the same accession (**P < 0.01). Error bars represent the standard deviation of three biological replicates (5 seeds each). (D) Kendall's tau‐b correlation between ROS levels and germination profile of A and R dry seeds from the yellow (Y1, Y2, Y3, Y4) and green (G1, G2, G3, G4) accessions

Seeds of four yellow pea (*Pisum sativum* L.) accessions (PIS 2, PIS 8, PIS 15 and PIS 224) and four green pea accessions (PIS 686, PIS 706, PIS 783 and PIS 2865) were provided by the Genebank Department of the Leibniz Institute of Plant Genetics and Crop Plant Research (IPK), Gatersleben (Germany). Recent seed viability monitoring of these accessions, carried out by IPK Genebank staff, pointed out differences in germination among seed lots stored in room conditions. Yellow and green accessions were renamed Y1, Y2, Y3, Y4 and G1, G2, G3, G4, respectively (Figure 1A; Table S1). Among these, the G4 accession was characterized by a wrinkled seed phenotype and classified as mutant (Figure 1A, G4; Table S1). More precisely, the G4 accession is a mutant of the variety named ‘Frogel’ (Auld et al., 1988). Although this mutant still needs to be thoroughly investigated at the genetic level, it is known that the wrinkled phenotype is frequently associated with the *r* (*rugosus*) locus in the *rr* configuration (Bhattacharyya et al., 1990; Rayner et al., 2017). The stored seeds used in this study were harvested in 2001. For each accession, fresh (F) seeds harvested in 2019 were also analysed as a proxy of how freshly harvested, non‐aged seeds should respond. All seed lots employed in the experiment (harvested both in 2001 and 2019) were grown at the experimental fields of IPK in Gatersleben, Germany (latitude 51° 49′ 19.74” N, longitude 11° 17′ 11.80″ E, 110.5 m.a.s.l., black soil of clayey loamy type). Seeds harvested in 2001 were transferred to a drying chamber (22 ± 2°C, 11 ± 3% relative humidity). After 3 weeks, samples were split into two different lots: seed lot A was kept in a cold chamber under controlled conditions (−18 ± 2°C, 8 ± 2% seed moisture content) and seed lot R was conserved at room temperature conditions (20 ± 2°C, 9 ± 2% seed moisture content). A germination test was performed in 2005 for all the study accessions (except for Y3), using the R seed lots. One replicate of 100 seeds per accession was employed in these tests; seeds were germinated between two layers of filter papers at a photoperiod of 14 h and a constant temperature of 25°C on a Jacobsen apparatus (RUMED). Germination ranged from 90% to 100%, data are provided in Table S1. In the current experiment, 30 seeds per replicate were ground and analysed using a moisture meter (Precisa XM 66), to determine the moisture content. For each seed lot, three replicates of 20 seeds were sown in Petri dishes with 1% agar as substrate and then placed in a growth chamber (22 ± 2°C, 70–80% RH, 150 μmol m^−2^ s^−1^ photon density, 16/8 h photoperiod). Petri dishes were checked for germination every 12 h for 14 days, and seeds were scored as germinated once the radicle had reached 2 mm in length. Germination was evaluated using the following parameters: *G* (germinability), *MGT* (mean germination time), *MGR* (mean germination rate), and *Z* (synchronization index), as described by Ranal & Garcia de Santana (2006) (Table S2).

### 2.2 ROS detection

The fluorogenic dye 2′,7′‐dichlorofluorescein diacetate (DCF‐DA; Sigma‐Aldrich) was used to quantify ROS levels released from dry seeds. Following deacetylation by cellular esterases, the dye is converted to a non‐fluorescent molecule which is subsequently oxidized by ROS into the highly fluorescent 2′,7′‐dichlorofluorescein (DCF). The assay was carried out as described by Forti et al. (2020), with the following modifications. Dry seeds (five seeds per replicate) were incubated in the dark for 30 min with 500 μl of a 10 μM DCF‐DA solution. Subsequently, three replicates (50 μl each) per seed lot were pipetted into 0.2 ml tubes and the emitted fluorescence was measured using the green channel (510 ± 5 nm) of a Rotor‐Gene 6000 PCR apparatus (Corbett Robotics), after a single cycle of 30 s at 25°C. As a negative control, three replicates containing only DCF‐DA were used to subtract the baseline fluorescence. Relative fluorescence was calculated by normalizing samples to controls and on the seed mass, then expressed as Relative Fluorescence Units (R.F.U.).

### 2.3 Determination of MDA levels

Malondialdehyde (MDA) levels were quantified as reported (Doria et al., 2019). For each accession, 30 dry seeds were ground in a Retsch Mixer Mill M 301 (RetschAllee) three times for 30 s at the vibrational frequency of 30 Hz s^−1^. For each lot, the resulting powder was divided into three replicates (0.2 g each) that were resuspended with 5 ml of H_2_O:0.5 M HClO_4_ solution (4:1) with 2% BHT (butylated hydroxytoluene, Sigma‐Aldrich) in ethanol to precipitate proteins. Samples were subsequently centrifuged (4°C, 10 min). MDA was determined as a thiobarbituric acid reactive substance (TBARS), following its reaction with thiobarbituric acid (TBA, Sigma‐Aldrich) at high temperature. For each sample, an aliquot of 100 μl was mixed with 100 μl of TBA in 1 ml dH_2_O and the mixture was heated in a boiling water bath at 95°C for 60 min. Test tubes were cooled at room temperature and absorbance was measured at 254 nm using a UV–visible spectrophotometer (UV‐1800, Shimadzu). A standard MDA (Sigma‐Aldrich) solution (100 μl, in a range of 0.025–0.1 mg ml^−1^) was added to a 1 ml test tube and mixed with TBA (100 μl) as previously described (Figure S1A).

### 2.4 Extraction and analysis of tocopherols

The extraction procedure was performed as described by Doria et al. (2019). For each accession, 30 dry seeds were ground as previously described. For each lot, an aliquot (0.5 g) of seed powder was added to 5 ml of ethanol containing 0.1% butylated hydroxytoluene (BHT, Sigma‐Aldrich) and the mixture was incubated for 10 min at 85°C. Subsequently, samples were subjected to saponification by adding 150 μl of 80% KOH and incubated for 10 min. After adding 3 ml of H_2_O, samples were placed on ice for 3 min, and then 3 ml of pure hexane were added. After shaking for 10 min at 800 rpm and centrifuging at 13,300 *g* the upper phase was transferred into a separate test tube, and the pellet was re‐extracted using 2 ml hexane. The combined hexane fractions were washed with 3 ml of dH_2_O, vortexed, centrifuged for 10 min and transferred into another test tube. Hexane fractions were dried using a vacuum evaporator, and the residue dissolved in 200 μl acetonitrile:methanol:dichloromethane 45:20:35 (v/v/v) prior to injection into an HPLC system (Kontron Instrument 420 system, Kontron Instruments) equipped with a C18 column (Zorbax ODS column 250 × 4.6 mm, 5 μ, Agilent Technologies). The isocratic mobile phase consisted of acetonitrile: methanol (60:40) (v/v), the flow rate was 1.0 ml min^−1^ at room temperature and absorbance was measured at 220 nm. As standard, γ‐tocopherol (Sigma‐Aldrich) was used for a calibration curve and identified in the chromatogram (Figure S1b).

### 2.5 Determination of free proline content

Free proline content was measured as described by Ábrahám et al. (2010) with the following modifications. For each accession, 30 dry seeds were ground as previously described. The seed powder (0.1 g) was added to 500 μl of 3% sulfosalicylic acid (Sigma‐Aldrich). Following centrifugation at 16,100 *g* for 5 min, a 100 μl aliquot of the extract was added to 500 μl of 3% sulfosalicylic acid: glacial acetic acid: acidic ninhydrin (1:2:2) (v/v/v). The reaction of ninhydrin with free proline was carried out at 96°C for 60 min and stopped on ice. Samples were then extracted with 1 ml of toluene. After 20 s vortex, phases were allowed to separate. The upper phase was transferred to quartz cuvettes and absorbance was read at 520 nm using a UV–visible spectrophotometer (UV‐1800, Shimadzu) and toluene as reference. A standard proline solution (100 μl, in a range of 0.001–0.1 mg ml^−1^) was prepared, added to a 2 ml test tube and mixed with the ninhydrin solution as previously described (Figure S1c).

### 2.6 Spectrophotometric determination of reducing sugars

The content of reducing sugars was measured as described by Miller (1959). For each accession, 30 dry seeds were ground as previously described. The seed powder (0.5 g) was added to 5 ml of dH_2_O, vortexed and incubated 2 h at 80°C in a water bath. After centrifugation at 1500 *g* for 15 min, the upper phase was transferred to new test tubes and the content of reducing sugars was quantified using DNS (3,5‐dinitrosalycilic acid, Sigma‐Aldrich) solution. Absorbance was read at 540 nm using a UV–visible spectrophotometer (UV‐1800, Shimadzu) and dH_2_O as reference. Standard solutions of glucose in the range of 0.4–1.5 mg ml^−1^ were used (Figure S1d).

### 2.7 Thermogravimetric analysis and differential scanning calorimetry

Thermal stability of the pea seed biomass and its content in organic substances were investigated by thermogravimetric analysis (TGA) under an air or nitrogen environment, respectively, using a Mettler Toledo TGA 1 instrument with a fixed heating rate of 20°C min^−1^. The temperature range was from 25°C to 800°C with a gas flow (air or nitrogen) of 4 l h^−1^ in the oven. For each accession, 30 dry seeds were ground into powder and sieved to a size of 100 μm. About 5 mg of sample was used in each test. This technique allows determination of the mass variation of the sample by heating and, depending on the starting and final temperature, each mass loss step can be attributed to the decomposition of the different components found in the sample. Differential scanning calorimetry (DSC) measurements allow studying the temperature and enthalpy associated with both first‐order transitions (namely, the physical change of state of the materials) and second‐order transitions (e.g. glass transition temperature *T*_g_, the temperature at which polymers undergo a transition from a brittle, glassy state to a rubbery state). Analyses were performed using a Q2000 Instrument (TA Instruments) interfaced with a TA5000 data station, using the two following procedures: (1) cooling the ground powders (approximately 5 mg, weighted in an open Al crucible) under nitrogen flux (50 ml min^−1^) from room temperature to −80°C, heating to 150°C, cooling the samples down to −80°C and heating to 25°C (all the ramps at a rate β = 5°C min^−1^); this allowed a first evaluation of the possible physical transitions of the different components in the starting material; (2) heating under nitrogen flux (50 ml min^−1^) from room temperature to 50°C, appending an isothermal stage of 12 h, cooling the samples down to −80°C, heating to 150°C, and finally cooling to room temperature (all the ramps at a rate β = 5°C min^−1^). Procedure (2) was conducted to remove the hydration water (by the isothermal stage) and measure the glass transition temperature *T*_g_. For each sample, three independent measurements were carried out. The temperature accuracy of the instrument was ±0.1°C, the precision was ±0.01°C, and the calorimetric reproducibility was ±0.05°C. The calorimetric profiles were analysed by the Universal Analysis software (TA Instruments), and the *T*_g_ values were determined using the suitable tool of the same software that considers the signal as a step and *T*_g_ as its inflection point.

### 2.8 Nuclear staining with toluidine blue

Embryos excised from four dry seeds of Y1 and G4 accessions, respectively, were fixed with 2% paraformaldehyde/0.2% glutaraldehyde (Sigma‐Aldrich) for 3 h at 4°C. Embryos were rinsed in phosphate‐buffered saline (PBS, pH 7.2) overnight, and then incubated in 0.5 M NH_4_Cl for 30 min at room temperature. Semithin sections (500 nm in thickness) were cut using an ultramicrotome, embedded in acrylic LR‐White resin (Agar Scientific) and allowed to harden at 60°C overnight. Toluidine blue staining was performed by covering the tissue sections, prepared as previously described, with a drop of the dye and incubating for 5 min at 100°C. Sections were then washed thoroughly with dH_2_O to remove dye excess, air‐dried, mounted in Mowiol (Sigma Aldrich) and finally imaged using Zeiss Axioskop 2 plus microscope. Nuclear and heterochromatic areas were measured from 10 nuclei per accession using ImageJ (Schneider et al., 2012) and heterochromatic areas were expressed as a percentage over the nuclear area.

### 2.9 Immunodetection of γH2AX foci

In order to detect the occurrence of γH2AX foci in the nucleus, sections prepared as previously described were subjected to the indirect immunohistochemical reaction by incubating them with the primary antibody Phospho‐Histone H2A.X (Ser139) Polyclonal Antibody from rabbit (ThermoFisher Scientific) according to the supplier's suggestions and subsequently with a secondary antibody coupled with 12 nm colloidal gold grain. Sections were stained by EDTA regressive technique (Bernhard, 1969) and observed with a Jeol JEM‐2100Plus electron microscope equipped with a 30 mm objective aperture and operating at 80 kV. Images were submitted to morphometric analyses using the software ImageJ (https://imagej.nih.gov/ij). The results are expressed as mean values ± SEM. For each sample, 10 nuclei were scored for the presence of γH2AX foci. The density of foci was calculated as follows: 100 squares (each one with an area of 400 nm^2^) were identified and the number of foci per single area was counted. The measurement was performed considering 10 cells for each sample and 10 squares per single cell.

### 2.10 Statistical analysis

Statistical analysis was performed in IBM SPSS 21.0 and the R environment for statistical computing and graphics (studio version 4.0.2). The following packages were used: *plyr* (Wickham, 2011), *ggplot2* (Wickham, 2016), *corrplot* (Wei & Simko, 2017), *ppcor* (Kim, 2015), *multcomp* (Hothorn et al., 2008), and *lsmeans* (Lenth, 2016). After checking data for normality and homoscedasticity, generalized linear models (GLM) were applied to evaluate the effect of accession, type of conservation, their interaction on different variables (germination parameters, ROS levels, chlorophyll content, tocopherols, MDA, free proline, reducing sugars); post‐hoc Tukey's or Bonferroni tests were used to perform multiple comparisons. A two‐way ANOVA was used to determine the effect of accession and conservation on the temperature of glass transition (*T*_g_). Correlations were performed with Pearson or Kendall's Tau‐b tests. Partial correlations were computed to evaluate the correlation between ROS content and germination percentage controlling for the effect of other variables (MC, Accession, Conservation). Statistical analyses to compare the percentage of heterochromatic areas and quantify the occurrence of γH2AX foci in the nucleus were performed using the two‐tailed paired Student's *t*‐test.

## 3 RESULTS

### 3.1 Germination tests reveal genotype‐dependent changes in pea seed longevity

Germination percentages of seeds stored under different environments (A and R) were analysed and compared (Figure 1B), whereas fresh seeds (F) were also included in the study, as proxy for the response expected from non‐aged samples (Figure 1B). Significantly lower germination percentages were observed in R seeds when compared to A (*P* < 0.001), except for the G4 variety (*P* = 1). The latter showed nearly 100% germination in A and R conditions, indicating the lack of viability decline during the 20 years‐storage periods (Figure 1B, Table S3). In all the tested accessions, the freshly harvested seeds (F) revealed a germination response similar to that observed in seeds stored under controlled conditions (A). Accessions and storage conditions (F, A and R) had a statistically significant effect on germination percentage and *MGT*, as did the accession × conservation interaction (*P* < 0.01) (see Table S4 for the corresponding Wald χ‐squared values and d.f.). A significant increase in *MGT* was observed for R seeds of all the accessions when compared to their A counterparts (*P* < 0.01) and to F seeds (*P* < 0.01), except for G4, which did not show differences among the two conditions (*P* = 1). Overall, *MGR* showed significantly higher values in A and R seeds than F, while *Z* did not show significant differences among accessions or conservation conditions (Tables S3 and S4). In order to figure out any possible effects related to chlorophyll content on seed germination, chlorophyll *a* and *b* levels were measured in dry and imbibed seeds of the green pea accessions (Figure S2). No significant correlation was found between chlorophyll *a* and *b* total contents or their ratio and germination percentage (Figure S3). However, a significantly lower chlorophyll *a* and *b* content was observed in the G4 variety compared to G1 and G3 (*P* < 0.01), while no difference was observed in terms of ratios. Moreover, R seeds of the G2 and G4 accessions did not show significant differences in the chlorophyll *a* and *b* contents, but they were significantly lower when compared to the R seeds of G1 and G3. Therefore, no clear pattern of association was observed between the chlorophyll content and the germination in aged green pea seeds, even though we cannot exclude that a lower content of chlorophyll in G4 could contribute to its higher longevity.

### 3.2 ROS levels and impact of oxidative stress in stored pea seeds

Different cellular parameters, usually affected by ageing, were analysed in the yellow and green pea accessions. ROS levels, as a measure of oxidative stress conditions, were determined first (Figure 1C). R seeds showed a significantly (*P* < 0.05) higher ROS content than A seeds in all the tested accessions, except for the long‐lived G4. The latter showed similar ROS levels in A and R seed lots (Figure 1C). Overall, ROS content in stored seeds (both A and R) showed a negative correlation with germination percentage (Tau‐b: −0.76; Figure 1D). In order to analyse this correlation by controlling for the effect of MC, accession and conservation, a partial correlation was computed. The negative correlation was confirmed (Tau‐b: ‐0.52, *P* < 0.01). ROS contents detected in pea seeds at 24 and 48 h of imbibition (Figure S4A) showed a negative correlation with germination percentage (Tau‐b: −0.57, −0.59, respectively; Figure S4B,C). Accessions, storage conditions (A and R) and their interaction had a statistically significant effect on ROS content (*P* < 0.01) (See Table S4 for the corresponding Wald χ‐squared values and d.f.). Based on the reported germination profiles and ROS levels, four pea accessions were selected for the biochemical analyses: the long‐lived G4 seeds, the G1 and Y1 accessions (showing intermediate and low germination in R seeds), and the Y2 accession (R seeds failed to germinate).

**FIGURE 2:**
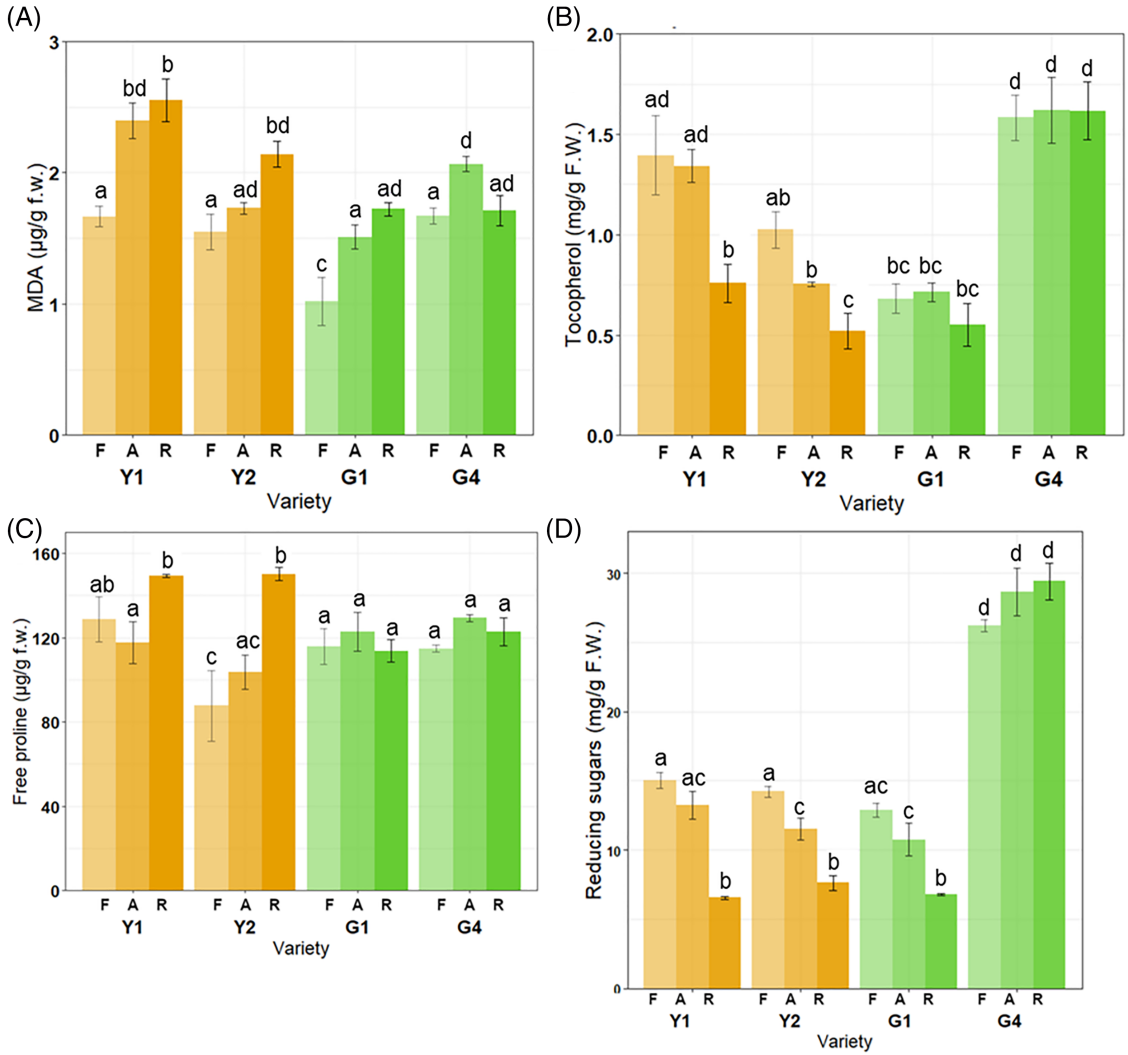
Levels of malondialdehyde (MDA) (A), tocopherols (B), free proline (C), and reducing sugars (D) in dry seeds of the yellow (Y) and green (G) pea accessions. F, harvested in 2019 (fresh). A, harvested in 2001 and kept in cold storage (aged). R, harvested in 2001 and conserved at room temperature conditions. Fresh seeds, collected from plants of the different accessions regenerated at IPK in 2019, were included only as a proxy of the behavior of freshly harvested, non‐stored seeds. Different letters above bars represent statistically significant differences in the content of target compounds

These accessions were tested for oxidative damage expressed as MDA content (a hallmark of lipid peroxidation), total tocopherols (indicative of the seed antioxidant response), free proline and reducing sugars content (hallmarks of seed deterioration; Figure 2). MDA levels were significantly higher in the R seeds of Y2 compared to the A seeds (*P* < 0.01), the accession that failed to germinate when conserved at room conditions. In the long‐lived G4 accession, similar MDA contents were detected in the stored seeds (R, 1.7 ± 0.11 μg/g_FW_; A, 2.07 ± 0.06 μg/g_FW_) and these values resembled the content found in F seeds (1.66 ± 0.06 μg/g_FW_), used in this investigation as a proxy of the response displayed by freshly harvested seeds (Figure 2A).

In the tested accessions, a significant decrease in terms of total tocopherols content was observed in R seeds compared to A seeds in the Y1 and Y2 accessions (*P* < 0.01), while G1 and G4 did not show differences in terms of tocopherols content among the A and R conditions (*P* = 1). When considering the response of freshly harvested seeds, the estimated tocopherols content in the Y1, Y2, G1 and G4 accessions ranged from 680 ± 70 to 1580 ± 110 μg/g_FW_ (Figure 2B).

Proline accumulation, typically observed *in planta* under oxidative stress conditions, has also been reported during prolonged seed storage (Kong et al., 2015). Proline levels were significantly higher only in R seeds of Y1 (*P* < 0.01) and Y2 (*P* < 0.01), compared to A (Figure 2C). F seeds showed proline contents in the 87.68 × 10^3^ ± 16.84 × 10^3^–128.69 × 10^3^ ± 10.61 × 10^3^ μg/g_FW_ range. Considering the documented role of reducing sugars in seed deterioration (Murthy & Sun, 2000), the levels of these metabolites were measured in the Y1, Y2, G1 and G4 accessions. A significant (*P* < 0.01) decrease in the content of reducing sugars was observed in the R seeds of Y1, Y2, and G1 accessions, compared to A seeds. In G4, the reducing sugars content of A seeds (28.61 × 10^3^ ± 1.72 × 10^3^ μg/g_FW_) was similar to that found in R seeds (29.40 × 10^3^ ± 1.32 × 10^3^ μg/g_FW_) and these levels were significantly higher than those observed in the other accessions. As for F seeds, the levels of reducing sugars varied from 12.88 × 10^3^ ± 0.51 × 10^3^ μg/g_FW_ (G1) to 26.19 × 10^3^ ± 0.41 × 10^3^ μg/g_FW_ (G4) Accessions, storage conditions (F, A, R) and their interaction had a statistically significant effect on all the tested metabolites (Table S5). A negative correlation with germination was observed for MDA content (Tau‐b: ‐0.52; *P* < 0.01) and free proline levels (Tau‐b: ‐0.61; *P* < 0.01) whereas a positive correlation was evidenced for total tocopherols content (Tau‐b: 0.58; *P* < 0.01) and reducing sugars (Tau‐b 0.58; *P* < 0.01). Correlation plots are available in Figure S5. Overall, the metabolites hereby tested as hallmarks of oxidative damage and antioxidant response show accumulation levels aligned with the germination phenotype and seed viability of the different pea accessions, indicating the impact of ageing at the cellular level. Despite ageing, limited oxidative damage was detected in the G4 seeds, and this finding is consistent with the observed long‐lived phenotype. As previously indicated, analyses on the F samples were included in the study to represent the response provided by freshly collected seeds.

### 3.3 Thermal decomposition analysis suggests the presence of low molecular weight components in the G4 seeds

Moisture content was measured in all the pea accessions, for the different conservation conditions (A, R), and in fresh (F) seeds (Table S3; Figure S6). The accession × conservation interaction showed a significant effect on moisture content, and the R seeds of G4 showed a significantly lower MC compared to the R seeds of the other accessions (Table S4, Figure S7). Thermoanalytical measurements were performed on all the eight pea accessions. Glass transition temperatures (*T*_g_) were measured using differential scanning calorimetry (Figure S6). By heating from −80 to 150°C (see Section 2), all the tested samples showed in the calorimetric profile an endothermic peak due to moisture release (Figure S7A, S7). This finding was confirmed by thermogravimetric analysis, which showed a first mass loss step (namely a decrease in the mass) by heating from room temperature to 130°C (Figure S8). In order to study the physicochemical properties of the components of the pea seeds, samples were treated at 50°C for 12 h to allow water release (first heating and isothermal step, see Section 2, procedure b) before cooling and subsequent heating up to 150°C. Samples were subsequently heated in the calorimetric instrument. Upon this last heating ramp, only one step underlying a change in the heat flux, and hence in the thermal capacity of the materials, was evident in the calorimetric curve in the temperature range from −80 and 150°C (Figure S7B, S7). This means that no chemical reactions took place in the dried samples, whereas only a transition in the physical state (from glassy to rubbery) of the polymers present in the seeds was detected. As shown in Table S6, even though *T*_g_ was significantly different among accessions (*F* = 28.634; df = 3; *P* < 0.01) and conservation states (F = 6.597; df = 2; *P* < 0.01), varying in the range 69–80°C, and underlying differences among the physicochemical properties of the samples, the values were not significantly correlated with germination percentage (Tau‐b = − 0.04, *P* = 0.806). Such glass transition temperatures, according to the Flory‐Fox equations, are characteristics of polymers with molar mass in the range 2500–3000 g mol^−1^ (Fox Jr & Flory, 1950). Since the glass transition was due to the re‐arrangement of the polymer chains, with no involvement of mass loss, its presence could not be revealed by TGA analyses.

**FIGURE 3:**
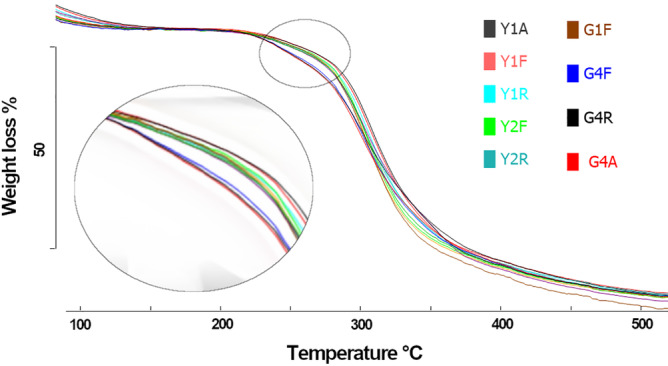
Weight loss (%) TG curves for dry seeds of the yellow (Y) and green (G) pea accessions

Moisture content was also evaluated by thermogravimetric analysis (Figure 3). The moisture content of the dry pea seeds was 10 ± 1%, visible as a weight‐loss close to 100°C. TGA measurements allowed for following the decomposition of the samples upon heating and for distinguishing the different reaction steps as a function of temperature, attributing them a decomposition percentage. The separation between the steps is better evidenced by considering the derivative curve of the mass loss profile, called DTG, given by the software. For all the samples, the moisture release step is evident from room temperature to around 130°C by a step in the TGA curve and a peak in the DTG signal. Interestingly, at higher temperatures, under a nitrogen atmosphere, the thermal decomposition profile of all the samples shows two subsequent steps: the first one occurring close to 250°C (as evident by the DTG curve as a shoulder) and the second one, more evident, at 300°C (evident as a peak in the DTG). In order to better evidence the differences in composition between seeds, curves were translated to match the same dry weight at 170°C. This point was chosen because at this temperature the loss of water was completed, and thermal decomposition had not started yet. As shown in Figure 3 and Figure S7, it is evident that the curves corresponding to the G4 seeds in the different tested conditions started to lose material close to 200°C. This is indicated by the different TGA curve slopes observed for the G4 seeds in the range of 200–300°C. This profile might correspond to the decomposition and/or evaporation of relatively small molecules exclusively found in the wrinkled seeds. The reported TGA curves suggested a particular biochemical composition of G4 seeds. Such a finding might be correlated to the wrinkled seed phenotype observed in the mutant G4 accession.

### 3.4 PAS‐TEM analysis reveals the presence of low molecular weight oligosaccharides in the R seeds of the long‐lived G4 accession

In order to gain further insights on biochemical features of G4 seeds that might correlate with the results of TGA analysis, Periodic acid‐Schiff (PAS) staining combined with transmission electron microscopy (TEM) was used to localize the occurrence of polysaccharides in the pea embryo axes. The progression of the oxidation reaction within polysaccharides, and the consequent generation of aldehyde groups, is delayed in these high molecular weight polymers, with the occurrence of dark‐stained dots. In contrast, oxidation is accelerated when low molecular weight oligosaccharides are the predominant substrates, resulting in staining of weak intensity. The PAS‐TEM analysis was carried out on R seeds of the G4 and Y1 accessions, harvested in 2001 and conserved at room temperature conditions. As for the G4 seeds, in the cytoplasm of cell embryos, the PAS reaction highlighted the occurrence of dots of low intensity (Figure S10A,B), whereas dark‐stained dots were observed in the PAS‐treated Y1 cells (Figure S10A). Dark dots reflect the ongoing oxidation of polysaccharides in the Y1 cells, while the poor signal detected in the G4 sample indicates that the same process was already concluded. The different reactivity hereby observed might reflect different carbohydrate composition profiles in the seeds of the two pea accessions, particularly the occurrence of low molecular weight oligosaccharides in the G4 seeds, as suggested by the TGA measurements.

### 3.5 Nuclear architecture and DNA damage levels in aged seeds

**FIGURE 4:**
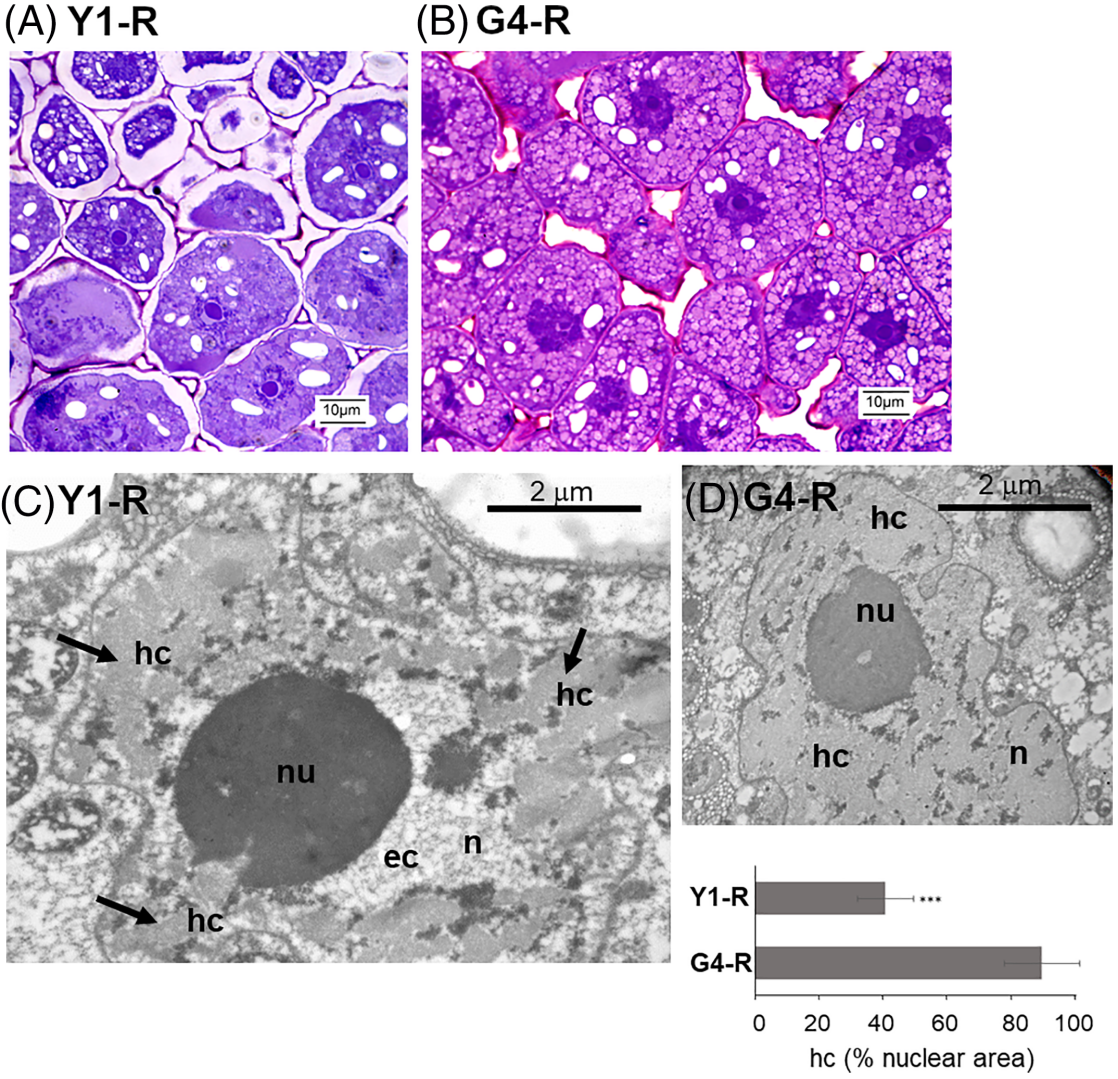
Nuclear morphology in embryo axes excised from R seeds of Y1 and G4 accessions with contrasting longevity. (A) Results of staining with toluidine blue performed on R seeds of the Y1 accession (B) Results of staining with toluidine blue performed on R seeds of the G4 accession. (C) Ultrastructural changes in chromatin distribution highlighted in Y1 nuclei subjected to EDTA regressive staining for TEM analysis. (D) Ultrastructural changes in chromatin distribution highlighted in G4 nuclei subjected to EDTA regressive staining for TEM analysis. (E) Percentage of heterochromatin per nuclear area. hc, heterochromatin; ec, euchromatin; nu, nucleolus; n, nucleus. ***P < 0.001

The previously reported biochemical data evidenced the contrasting impact of ageing, in terms of oxidative damage, in the long‐lived G4 seeds compared to Y1, Y2, and G1 accessions. To assess whether the observed oxidative stress profiles were associated with changes in nuclear ultrastructure and DNA damage levels, TEM‐based analyses were carried out. This investigation was restricted to embryos excised from R seeds of the G4 and Y1 accessions, both subjected to the same ageing conditions but bearing significant differences in the cellular oxidative stress hallmarks. Preliminary nuclear staining with Toluidine Blue carried out on sections of pea embryo axes revealed distinctive nuclear morphologies. The Y1 nuclei showed a compacted nucleolus (Figure 4A, nu, arrow) surrounded by dark blue stained areas corresponding to heterochromatin domains (Figure 4A, hc, arrow). Such morphology, recurrent in Y1 embryos, differed from that observed in G4 embryos. As shown in Figure 4B (n), each G4 nucleus of the section contained larger and more uniform dark blue stained areas, suggesting an expansion of the heterochromatin regions. Such morphologies were further evidenced by TEM analysis. In the Y1 nuclei, large heterochromatin areas are visible (40.75 ± 8.77% of the nuclear area), as expected when dehydration occurs (Figure 4C,E, hc, arrows), as well as regions of decondensed euchromatin (Figure 4C, ec). Some of these condensed heterochromatin regions are located close to the nucleolus. In the G4 nuclei, chromatin condensation patterns were remarkably enhanced, covering the vast majority (89.69 ± 11.84%) of the nuclear area (Figure 4D,E, hc). Morphological profiles were then combined with the investigation of the γH2AX foci distribution.

**FIGURE 5:**
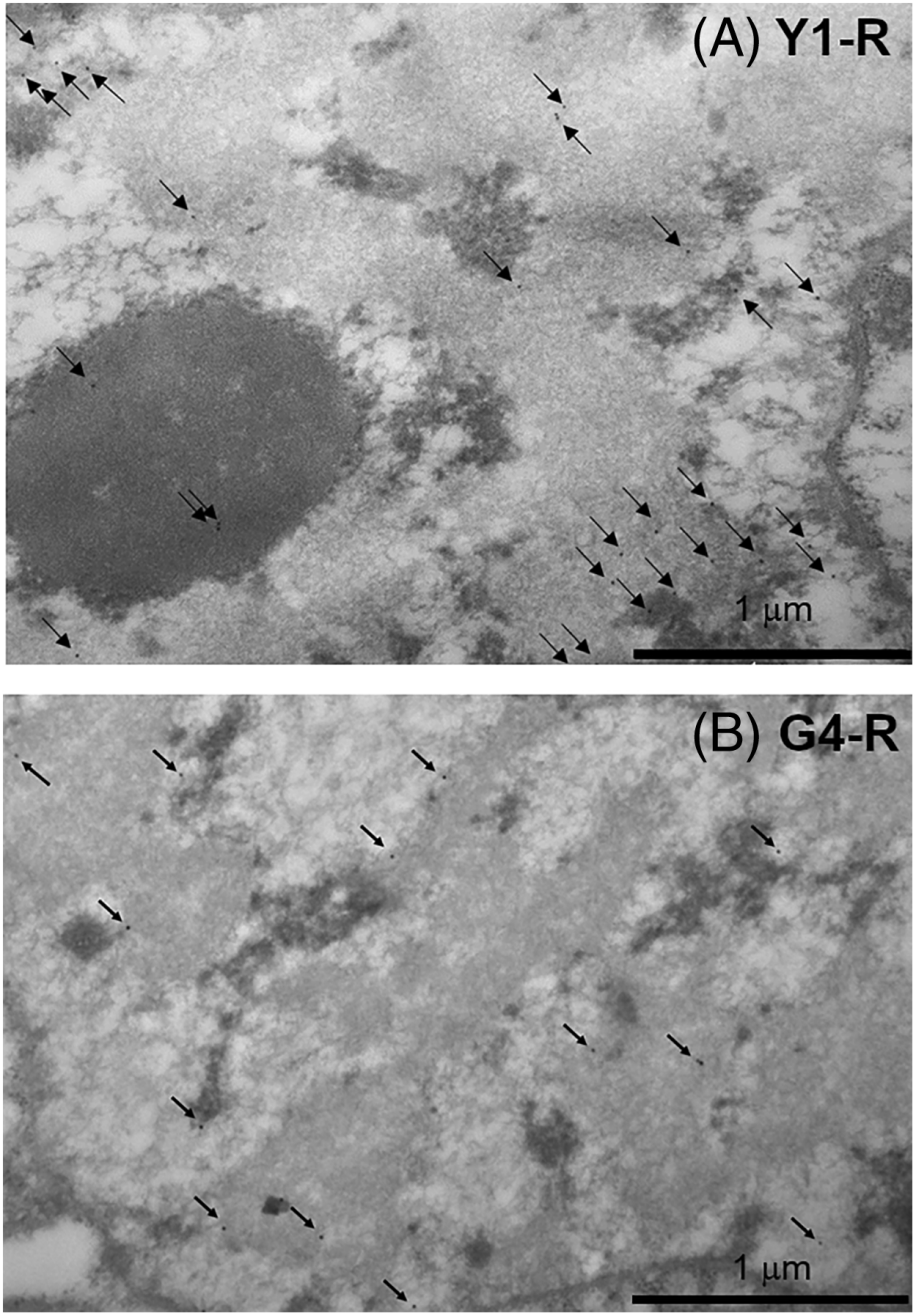
Distribution of γH2AX foci in the nucleus of embryo axis excised from seeds stored at room temperature: (A) Y1‐R and (B) G4‐R. Detection of γH2AX foci was performed by immunocytochemical and TEM analyses. n, nucleus; nu, nucleolus.

Upon DNA damage, ATM phosphorylates the histone variant H2AX on Ser139 (Burma et al., 2001) and such modification (γH2AX) can spread for up to 1 Mb away from the break site, acting as a platform to recruit the DNA repair enzymes (Iacovoni et al., 2010). In order to map the γH2AX foci in the Y1 and G4 pea nuclei, immunocytochemical and TEM analyses were performed, using an antibody raised against the human histone variant H2AX on Ser139. Representative examples of the distribution of γH2AX foci in the nuclei of R seeds, in both Y1 and G4 accessions, are shown in Figure 5A,B. The estimated density of γH2AX foci, expressed as n° foci per 400 nm^2^ of nuclear area, was significantly higher (*P* < 0.05) in the Y1 nuclei (1.6), compared to G4 nuclei (1.1). Thus, the limited genotoxic impact exerted by long‐term storage on the wrinkled seeds might be the consequence of the highly compacted chromatin conformation previously described.

## 4 DISCUSSION

Assessing seed longevity of conserved accession is of key importance in genebank management in order to put in place an effective monitoring system that checks the viability status of stored samples at appropriate intervals (FAO, 2014). The pea accessions analysed in the present work provided a unique opportunity to investigate different physiological and molecular aspects of seed longevity under seed bank storage. Room temperature storage (20°C) of the tested pea accessions caused increased deterioration, with the exception of the G4 accession, when compared to controlled storage conditions (−18°C; Figure 1B). Based on the information provided by IPK Genebank, the G4 accession has been classified as a mutant of the ‘Frogel’ variety (Auld et al., 1988), showing a wrinkled seed phenotype and a unique example among the eight pea accessions investigated in the present study.

The comparative analysis of the eight pea accessions confirmed the inverse correlation between ROS levels and seed longevity. Fresh seeds, collected from plants of the different accessions regenerated at IPK in 2019, were also included in the study as a proxy of the behaviour of freshly harvested, non‐stored seeds. In studies on seed longevity of long‐termed stored accessions, it is very unlikely to have the same molecular and physiological data from both stored and freshly harvested seeds. Thus, in long‐term experiments on seed longevity, such as the one recently started at the Svalbard Seed Vault, it will be important to properly compare seed traits before and after storage (IPK, 2020). Even if all accessions and seed lots (harvested in 2001 and 2019) were regenerated in the same location (IPK, Gatersleben, Germany), we could not rule out that different environmental factors (mainly temperature and rainfall) experienced by the mother plants of the same accessions in different regeneration years, might have influenced different seed traits (Mondoni et al., 2014). Therefore, the study was mainly focused on the comparison between different storage conditions (A and R). As previously mentioned, the analyses on F seeds were only considered representative of the behaviour of freshly harvested seeds. Given this, in fresh seeds (F), high and uniform germination was observed in all accessions (Figure 1B). Moreover, all the accessions displayed ROS contents that varied within a limited range (Figure 1C). Indeed, ROS production in fresh seeds during post‐harvest storage has been documented (Bailly et al., 2008). The low ROS levels observed in seeds aged for about 20 years under controlled conditions (A) correlated with the high germinability profiles of all the investigated accessions (Figure 1D). The long‐term storage at room temperature conditions (R) caused significant ROS accumulation in all the accessions showing a drop in germination, except for G4 (Figure 1C). This finding poses a question in regard to the mechanisms underlying the ability of G4 seeds to control ROS levels. To address this issue, specific metabolites associated with the seed ability to scavenge the toxic free radicals were measured. The investigation was restricted to four pea accessions, namely, Y1, Y2, G1, and G4, showing contrasting germination and ROS profiles.

ROS‐driven oxidation targets polyunsaturated fatty acids, generating lipid peroxides (Gaschler & Stockwell, 2017). The G4 seeds displayed the highest levels of the lipophilic antioxidant tocopherols, known for their ability to scavenge lipid peroxyl radicals (Fritsche et al., 2017), independent of their conservation state (Figure 2B). Thus, it appears there was no need to exploit the tocopherols pool in G4 seeds since lipid peroxidation did not overcome a critical threshold. In the G1 accession, characterized by a low tocopherol content (approximately 50% less than G4), longevity was compromised following long‐term storage at room temperature conditions. Although tocopherols were utilized by the Y1 and Y2, this was insufficient to avoid the germinability drop in R seeds. Free proline is an efficient ROS scavenger and a compatible osmolyte (Hayat et al., 2012; Liang et al., 2013). Increased free proline content contributed to oxidative stress adaptation in oat (*Avena sativa* L.) seeds with higher moisture content, stored for up to 1 year (Kong et al., 2015). Similar free proline content was detected in G1 and G4 accessions in all the tested treatments, whereas a significant accumulation occurred in the Y1 and Y2 seeds stored under room temperature conditions (R) (Figure 2C). This finding corroborates for the first time the role of free proline as a seed‐specific oxidative damage marker during long‐term ageing under seed bank conditions. Based on the reported data, it is difficult to compare the observed ROS levels with the metabolite contents detected in the seeds of Y and G accessions stored under different conditions (A and R seeds, respectively). In A seeds of the different accessions showing minimal changes in ROS levels, the observed, significant fluctuations in those metabolites used as hallmarks of cellular damage (e.g. MDA, free proline; Figure 2A,C) were evidently compatible with seed viability. On the other hand, the depletion of metabolites exerting protective roles (e.g. tocopherols and reducing sugars; Figure 2B,D) detected in the non‐viable R seeds did not allow oxidative damage rescue.

As previously mentioned, G4 is a unique example of a wrinkled mutant among the eight pea accessions hereby analysed. It has been reported that wrinkled pea seeds carrying mutations at the *r* (*rugosus*) and *rb* (*ADP‐glucose pyrophosphorylase*) loci display alterations in the starch biosynthetic pathway resulting in pleiotropic effects such as accumulation of the raffinose family oligosaccharides (Gawlowska et al., 2017; Stickland & Wilson, 1983). Such a feature has also been associated with membrane stability (Crowe et al., 1992). Indeed, significantly higher levels of reducing sugars were detected in the fresh G4 seeds, whereas no decrease was observed in A and R seeds after long‐term storage, apart from what occurred in Y1, Y2 and G1 (Figure 2D). Reducing sugars that participate in non‐enzymatic protein glycosylation (Maillard reaction), together with lipid peroxidation, is indicative of the biochemical deterioration associated with seed ageing (Murthy & Sun, 2000). The low ROS content of G4 seeds prevented damage, whereas reducing sugars were engaged in the Maillard process triggered by the oxidative environment of Y1, Y2 and G1 seeds. This could also be linked to lower retention of chlorophyll in the mature seeds, as it has been postulated that incomplete chloroplast dedifferentiation can lead to oxidative damage. Alternatively, low chlorophyll contents limit the accumulation of phototoxic compounds derived from the chlorophyll degradation occurring during seed storage (Zinsmeister et al., 2016).

The research question that arises from the reported data is how the G4 seeds can maintain constitutive low ROS levels despite 20 years of storage, considering that ROS are continuously generated in an oxygenic environment and the activity of ROS scavenging enzymes is restricted in the glassy state of dry cytoplasm (Nagel et al., 2019). In this environment, the dry seed exploits the pool of available antioxidant compounds, as observed for the Y and G accessions. The study of mechanical properties within the dry cytoplasm of embryonic pea axes has revealed low molecular mobility over a broad range of moisture contents and temperatures, possibly due to steric hindrance between adjacent macromolecules, and that such features might contribute to seed longevity (Ballesteros & Walters, 2019). However, there is scanty information concerning the mechanical properties of the dry cytoplasm in wrinkled pea seeds and their possible role in longevity.

The TGA curves recorded in the G4 seeds, independent of treatments, suggested the presence of relatively small molecules (Figure 3), a finding corroborated by results of PAS‐TEM analysis pointing to the occurrence of low molecular weight oligosaccharides (Figure S9). At the moment, we cannot rule out the possibility that G4 seeds use specific low molecular‐weight antioxidant molecules, for example, glutathione, as a redox buffer to maintain ROS within a threshold critical to ensure longevity. It has been reported that mutations at the *r* locus altering seed composition and hygroscopic properties, can affect seed longevity (Lyall et al., 2003).

The ability to buffer ROS accumulation is expected to restrict genotoxic damage, but nuclear rearrangements might provide protection (Lee et al., 2020). In the G4 accession, almost the entirety of the nuclear area is filled with heterochromatin (Figure 4B,D). Chromatin condensation occurring after severe water loss might be promoted by increased levels of cations and changes in histone variants (Deltour, 1985; Washio, 2014). This aspect is still poorly explored in the context of seed longevity, and the G4 accession might provide a unique working system for further investigations of the dynamics of nuclear architecture in response to desiccation. At the moment, it is not possible to determine to what extent the mutation carried by the G4 accession might contribute to the observed nuclear architecture. According to Bhattacharyya et al. (1993), the high sucrose content of wrinkled pea seeds enhances the embryo osmotic potential, increasing water uptake during seed development. Subsequently, during desiccation excess water is lost, causing the wrinkled phenotype. These shrinkage dynamics might lead to a tighter chromatin conformation useful for genome maintenance (Bhattacharyya et al., 1993). Future studies focused on the G4 accession will provide a deeper understanding of the impact of their physic‐chemical properties on nuclear ultrastructure.

The G4 seeds aged at room temperature showed a significantly lower frequency of the γH2AX foci compared to the Y1 seeds (Figure 5). Chromatin compaction protects DNA from damage, but it also blocks the expansion of H2AX phosphorylation (Cann & Dellaire, 2011; Nair et al., 2017). The high‐longevity profile of G4 seeds and genotoxic stress resilience were possibly influenced positively by such chromatin dynamics. These findings agree with those described in both yeast and mammalian cells, revealing that the γH2AX foci at double‐strand break sites were formed at lower levels in heterochromatin, when compared to euchromatin (Cowell et al., 2007; Kim et al., 2007). On the other hand, the protective effect of chromatin compaction might be related to non‐histone chromatin proteins that physically shield the genomic DNA (Falk et al., 2008). Results from ultrastructural and immunohistochemical analyses should also be critically assessed, considering the effect of seed moisture content, which was lower for the G4 sample (7.87% for R seeds) than for the other samples (8.52%–8.81%). According to Harrington's rule, even a 1.0% decrease in water content might double longevity (Harrington, 1960), whereas van Zanten et al. (2011) showed that chromatin compaction is influenced by moisture content. At the moment, the possibility that the observed differences between G4 and Y1 seeds in nuclear shrinkage and γH2AX density might be due to a difference in moisture content cannot be ruled out. Overall, it should also be noted that for those accessions showing germination percentage <100%, the tested samples included viable seeds at different physiological stages as well as dead seeds. Ideally, individual seeds should undergo preliminary non‐destructive tests to distinguish the viable, aged or dead ones and to correlate each specific physiological condition with the information provided by subsequent destructive analyses. Kranner, Kastberger, et al. (2010) reported that infrared thermography could be used as a non‐invasive technique that discriminates the thermal profile of seeds with different viability levels upon water uptake. Various other improved methodologies for non‐destructive seed viability that are now available (Xia et al., 2019), could be used for dedicated studies on seed longevity.

The present work underlines the relevance of multidisciplinary approaches in seed longevity studies, particularly when addressing intra‐species variations in longevity. The case study hereby reported brings to the forefront the redox context and some aspects of the genotoxic stress response that contributes to the long‐lived pea seed phenotype. Investigating the biological bases of these intra‐species variations will be fundamental in order to identify groups of seed accessions with different longevity profiles and therefore plan seed bank activities by calculating more precise seed viability monitoring intervals (FAO, 2014).

## AUTHOR CONTRIBUTIONS

**Andreas Börner**, **Maraeva Gianella**, **Filippo Guzzon** and **Alma Balestrazzi** conceived and designed the study. **Maraeva Gianella**, **Enrico Doria**, **Daniele Dondi**, **Chiara Milanese**, **Lucia Gallotti** and **Lorena Zannino** performed the experiments. Alma Balestrazzi and **Maraeva Gianella** wrote the manuscript. **Enrico Doria**, **Daniele Dondi**, **Chiara Milanese**, **Andreas Börner**, **Anca Macovei**, **Andrea Pagano**, **Filippo Guzzon** and **Marco Biggiogera** reviewed the manuscript. All authors read and approved the final manuscript.

## Supporting information


**Appendix S1.** Supporting Information.

## Data Availability

The data that support the findings of this study are available from the corresponding authors upon reasonable request.
