# Supplementary material for: Physiological and molecular aspects of seed longevity: exploring intra‐species variation in eight Pisum sativum L. accessions
Source: Physiol Plant. 2022 May 18;174(3):e13698. doi: 10.1111/ppl.13698 (PMC9321030; doi:10.1111/ppl.13698)
Supplement: Supplementary file 1 — Appendix S1. Supporting Information. [file PPL-174-0-s001.docx]

**Supplementary Data**

**Physiological and molecular aspects of seed longevity: exploring intra-species variation in eight *Pisum sativum* L. accessions**

Maraeva Gianella, Enrico Doria, Daniele Dondi, Chiara Milanese, Lucia Gallotti, Andreas Börner, Lorena Zannino, Anca Macovei, Andrea Pagano, Filippo Guzzon, Marco Biggiogera, Alma Balestrazzi

**Methods**

*Determination of chlorophyll a and b content*

Chlorophyll *a* and *b* contents were assessed with a modified protocol from Cubas et al. (2008). Seeds were grinded in liquid nitrogen, then four replicates (0.1 g each) were added to 1.5 ml of N, N-dimethylformamide (DMF) and 0.5 g Na_2_CO_3_ to prevent the formation of pheophytin, and vortexed thoroughly. Samples were then incubated at 4°C under agitation in the dark for 4 h. Absorbance was measured at 647 and 664 nm using a spectrophotometer (Jasco Inc. Mary's Ct, Easton, MD, U.S.A.). Chlorophyll *a* and *b* contents were then calculated according to Wellburn (1994) and expressed as μg/g fresh weight.

Results of this analysis, carried on G1, G2, G3, and G4 seeds (both dry and imbibed) are shown in Supplementary Fig. S2. No differences in terms of chlorophyll *a* and *b* degradation rate were observed, and no correlation was found between chlorophyll *a* and *b* total contents or their ratio and germination percentage (Supplementary Fig. S3). The reported data exclude any possible effects related to chlorophyll content on seed germination in the tested accessions.

**References**

**Cubas C, Lobo G, Gonzalez M. 2008.** Optimization of the extraction of chlorophylls in green beans (*Phaseolus vulgaris* L.) by N,N-dimethylformamide using response surface methodology. *Journal of Food Composition and Analysis* **21**: 125-133.

**Wellburn AR. 1994.** The spectral determination of chlorophylls *a* and *b*, as well as total carotenoids, using various solvents with spectrophotometers of different resolution. *Journal of Plant Physiology* **144**: 307-313.

*PAS-staining for polysaccharides reveals differential reactivity in embryo cells of G4 and Y1 accessions*

Periodic acid Schiff (PAS) staining uses a periodic acid solution which oxidizes units within polysaccharides, generating aldehyde groups that react with the colourless Schiff's reagent and produce magenta colour (Zugibe et al., 1970). Embryos excised from dry seeds were fixed with 2% paraformaldehyde/0.2% glutaraldehyde (Sigma-Aldrich) for 3 h at 4°C. Embryos were rinsed in phosphate-buffered saline (PBS, pH 7.2) overnight, incubated in 0.5 M NH_4_Cl for 30 min at 4°C, dehydrated in progressively concentrated ethanol solution, embedded in acrylic LR-White resin (Agar Scientific, Stansted, UK) and allow to harden at 60°C overnight. Sections (80 nm in thickness) were cut using an ultramicrotome and transferred on 300 mesh nickel grids Formvar-carbon coated, hydrated for 5 min, oxidized with 0.5% periodic acid (30 min), and rapidly rinsed three times with H_2_O. Sections were stained with the Schiff reagent Osmium ammine B (Polysciences) for 30 min, and finally rinsed with H_2_O. Sections were observed using a Jeol JEM-2100Plus electron microscope equipped with a 30 mm objective aperture and operating at 80 kV.

In order to provide additional information on the carbohydrate content, periodic acid-Schiff (PAS) staining combined with transmission electron microscopy was used to localize the occurrence of polysaccharides in the pea embryo axes. The progression of the oxidation reaction within polysaccharides, and the consequent generation of aldehyde groups, is delayed in these high molecular weight polymers, featuring the occurrence of dark-stained dots. By contrast, oxidation is accelerated when low molecular weight oligosaccharides are the predominant substrates, resulting in staining of weak intensity. The analysis was carried out on sections of the G4 pea accession, characterized by high-longevity, whereas the Y1 accession, showing low germination performance was used for comparison. For both accessions, attention was focused on the R seeds, harvested in 2001 and conserved at room temperature conditions. The progression of the oxidation reaction was monitored after 30 min of incubation with the dye. As for the G4 wrinkled seeds, in the cytoplasm of cell embryos the PAS reaction highlighted the occurrence of dots of low intensity within structures possibly corresponding to proplastids (Supplementary Fig. S8, a and b). Organelles found in the cytoplasm of dry seeds are generally difficult to classify due to their immature and poorly hydrated state. Embryo proplastids can however contain carbohydrates (Harrison, 1977). No reaction was observed in control samples without periodic acid treatment (Supplementary Fig. S8, c). Dark-stained dots were observed within the proplastids in the PAS-treated Y1 cells (Supplementary Fig. S9, a) whereas no reaction was observed in control samples without periodic acid treatment (Supplementary Fig. S9, b). The dark staining reflects the ongoing oxidation of polysaccharides in the Y1 cells whereas the poor signal detected in the G4 sample indicates that the same process was already concluded. The different reactivity hereby observed might reflect for different carbohydrate composition profiles in the seeds of the two pea accessions. Particularly, this finding suggests for the predominant occurrence of low molecular weight oligosaccharides, whose hydrolysis should require a shorter time, in the G4 seeds. However, additional biochemical and molecular studies will be necessary to better assess such preliminary observations

**References**

**Harrison CR. 1977.** Ultrastructural and histochemical changes during the germination of *Cattleya aurantiaca* (Orchidaceae). *Botanical Gazette* **138**: 41-45.

**Zugibe FT. 1970.** Positive periodic acid-Schiff staining of acid mucopolysaccharides. *Histochemistry Journal* **2**:191-197.

**Tables**

**Supplementary Table S1** Main features of the eight *Pisum sativum* L. accessions investigated in this study. Y, yellow. G, green. In the last column on the right, historical germination data, captured on R seeds in 2005 and expressed as final germination percentage, are presented. NA, not available.

| **Seed**  **lot** | **Accession number (IPK)** | **Seed coat**  **colour** | **Accession name** | **Country of origin** | **Biological**  **status** | **G% in 2005** |
| --- | --- | --- | --- | --- | --- | --- |
| Y1 | PIS 2 | Yellow | - | Greece | Landrace | 94 |
| Y2 | PIS 8 | Yellow | Nordost Kleine Weiße | Germany | Improved Cultivar | 90 |
| Y3 | PIS 15 | Yellow | Kronenerbse | Germany | Improved Cultivar | NA |
| Y4 | PIS 224 | Yellow | Sperba | Germany | Improved Cultivar | 100 |
| G1 | PIS 686 | Green | Fügeder Erbse Monte Express | Unknown | Improved Cultivar | 92 |
| G2 | PIS 706 | Green | Mauthner-Fall Express | Germany | Improved Cultivar | 92 |
| G3 | PIS 783 | Green | Grüne Saxa | Germany | Improved Cultivar | 94 |
| G4 | PIS 2865 | Green | Frogel | Unknown | Mutant | 90 |

**Supplementary Table S2** Germination parameters used in this study as reported by Ranal and Garcia de Santana (2006). For each parameter, definition, formula, limits of measurement and unit are shown. N: total seed number; n_i_: number of seeds germinated at the i^th^ observation; t_i_: time at the i^th^ observation (hours); k: time at the end of the experiment; f_i_: relative frequency of germination; C_ni,2_ : combination of the seeds germinated in the time i, two together.

| **Parameter** | **Definition** | **Formula** | **Limits** | **Unit** |
| --- | --- | --- | --- | --- |
| *G* | Germinability | $\boldsymbol{G=}\frac{\boldsymbol{(}\sum_{\boldsymbol{i=1}}^{\boldsymbol{k}} \boldsymbol{n}_{\boldsymbol{i}}\boldsymbol{)*100}}{\boldsymbol{N}}$ | 0 ≤ *G* ≤ 100 | % |
| *MGT* | Mean germination time | $\boldsymbol{MGT=}\frac{\sum_{\boldsymbol{i=1}}^{\boldsymbol{k}} \boldsymbol{n}_{\boldsymbol{i}}\boldsymbol{t}_{\boldsymbol{i}}}{\sum_{\boldsymbol{i=1}}^{\boldsymbol{k}} \boldsymbol{n}_{\boldsymbol{i}}}$ | 0 < *MGT* ≤ k | Hours |
| *CVG* | Coefficient of velocity of germination | $\boldsymbol{CVG=}\frac{\sum_{\boldsymbol{i=1}}^{\boldsymbol{k}} \boldsymbol{f}_{\boldsymbol{i}}}{\sum_{\boldsymbol{i=1}}^{\boldsymbol{k}} \boldsymbol{f}_{\boldsymbol{i}}\boldsymbol{n}_{\boldsymbol{i}}}\boldsymbol{*100}$ | 0 ≤ *CVG* ≤ 100 | % |
| *MGR* | Mean germination rate | $\boldsymbol{MGR=}\frac{\boldsymbol{CVG}}{\boldsymbol{100}}$ | 0 < *MGR* ≤ 1 | hour**^-1^** |
| *Z* | Synchronization index | $\boldsymbol{Z=}\frac{\sum_{\boldsymbol{i=1}}^{\boldsymbol{k}} \boldsymbol{C}_{\boldsymbol{n}_{\boldsymbol{i}}\boldsymbol{,2}}}{\boldsymbol{C}_{\sum\boldsymbol{n}_{\boldsymbol{i}}\boldsymbol{,2}}}$ | 0 ≤ Z ≤ 1 | Unit-less |

**Supplementary Table S3** Values expressed as mean ± SD of the germination parameters and moisture content (MC) for the eight pea accessions in their three different states of conservation (F, fresh seeds harvested in 2019; A, seeds harvested in 2001 and kept in cold storage; R, seeds harvested in 2001 and conserved at room temperature). *G*, germinability. *MTG*, mean germination time. *MGR*, mean germination rate. *Z*, synchrony of the germination process. Y, yellow. G, green. NA, not applicable.

| **Seed**  **lot** | **State** | ***G* (%)** | ***MGT* (h)** | ***MGR***  **(day^-1^)** | ***Z***  **(unit less)** | **MC (%)** |
| --- | --- | --- | --- | --- | --- | --- |
| **Y1** | **F** | 100.00 ± 0.00 | 75.24 ± 6.41 | 0.00919 ± 0.00009 | 0.16 ± 0.04 | 7.09 ± 0.09 |
|  | **A** | 100.00 ± 0.00 | 83.23 ± 2.48 | 0.01401 ± 0.00039 | 0.16 ± 0.01 | 8.34 ± 0.03 |
|  | **R** | 11.11 ± 5.09 | 136.89 ± 13.72 | 0.00808 ± 0.00105 | 0.18 ± 0.17 | 8.81 ± 0.04 |
| **Y2** | **F** | 100.00 ± 0.00 | 70.60 ± 3.2 | 0.00933 ± 0.00015 | 0.24 ± 0.02 | 7.42 ± 0.05 |
|  | **A** | 90.00 ± 3.33 | 93.49 ± 5.89 | 0.01273 ± 0.00015 | 0.20 ± 0.07 | 7.30 ± 0.23 |
|  | **R** | 0 | NA | NA | NA | 8.61 ± 0.05 |
| **Y3** | **F** | 83.33 ± 3.33 | 85.95 ± 2.00 | 0.00963 ± 0.00005 | 0.15 ± 0.01 | 7.47 ± 0.09 |
|  | **A** | 100.00 ± 0.00 | 69.79 ± 1.81 | 0.01303 ± 0.00052 | 0.20 ± 0.05 | 8.08 ± 0.10 |
|  | **R** | 5.55 ± 3.84 | 109.46 ± 14.65 | NA | NA | 8.68 ± 0.02 |
| **Y4** | **F** | 98.33 ± 2.88 | 77.20 ± 6.23 | 0.00942 ± 0.00016 | 0.15 ± 0.05 | 7.34 ± 0.09 |
|  | **A** | 92.22 ± 5.09 | 107.96 ± 1.86 | 0.01295 ± 0.00021 | 0.32 ± 0.09 | 8.47 ± 0.02 |
|  | **R** | 2.22 ± 1.92 | NA | NA | NA | 8.52 ± 0.05 |
| **G1** | **F** | 100.00 ± 0.00 | 79.19 ± 6.52 | 0.01042 ± 0.00096 | 0.29 ± 0.05 | 7.34 ± 0.09 |
|  | **A** | 98.88 ± 1.92 | 113.63 ± 0.89 | 0.01252 ± 0.00004 | 0.16 ± 0.02 | 8.48 ± 0.04 |
|  | **R** | 41.11 ± 1.92 | 106.64 ± 7.36 | 0.0105 ± 0.00022 | 0.16 ± 0.03 | 8.59 ± 0.07 |
| **G2** | **F** | 100.00 ± 0.00 | 68.99 ± 5.17 | 0.01041 ± 0.00001 | 0.24 ± 0.04 | 7.36 ± 0.01 |
|  | **A** | 100.00 ± 0.00 | 105.96 ± 4.4 | 0.01321 ± 0.00017 | 0.13 ± 0.12 | 8.82 ± 0.01 |
|  | **R** | 20.00 ± 0.00 | 110.52 ± 16.79 | 0.00918 ± 0.00046 | 0.28 ± 0.32 | NA |
| **G3** | **F** | 100.00 ± 0.00 | 72.03 ± 1.21 | 0.00942 ± 0.00011 | 0.22 ± 0.07 | 7.22 ± 0.01 |
|  | **A** | 98.88 ± 1.92 | 114.82 ± 3.24 | 0.013 ± 0.00019 | 0.22 ± 0.21 | 8.79 ± 0.06 |
|  | **R** | 0 | NA | NA | NA | 8.46 ± 0.02 |
| **G4** | **F** | 100.00 ± 0.00 | 69.86 ± 2.27 | 0.01644 ± 0.00025 | 0.34 ± 0.08 | 6.97 ± 0.29 |
|  | **A** | 96.66 ± 3.33 | 96.67 ± 1.38 | 0.01245 ± 0.00016 | 0.19 ± 0.01 | 7.42 ± 0.02 |
|  | **R** | 98.88 ± 1.92 | 101.10 ± 4.2 | 0.01313 ± 0.00015 | 0.19 ± 0.07 | 7.88 ± 0.02 |

**Supplementary Table S4** Results of the generalized linear models (GLMs) performed on germination parameters, moisture content (MC), and ROS (reactive oxygen species) contents in the eight accessions, three conservation states. F, fresh seeds harvested in 2019. A, seeds harvested in 2001 and kept in cold storage. R, seeds harvested in 2001 and conserved at room temperature. *G*, germinability. *MTG*, mean time to germination. *MGR*, mean germination rate. *Z*, synchrony of the germination process. Y, yellow. G, green.

| **Parameter** | **Factors** | **Wald χ-squared** | **d.f.** | ***P* value** |
| --- | --- | --- | --- | --- |
| ***G*** | **Accession** | 1842.469 | 6 | <0.001 |
|  | **Conservation** | 146.002 | 2 | <0.001 |
|  | **Accession*Conservation** | 1482.498 | 14 | <0.001 |
| ***MTG*** | **Accession** | 71.755 | 6 | <0.001 |
|  | **Conservation** | 13.539 | 2 | <0.001 |
|  | **Accession*Conservation** | 97.988 | 14 | <0.001 |
| ***MGR*** | **Accession** | 148.311 | 6 | <0.001 |
|  | **Conservation** | 455.482 | 2 | <0.001 |
|  | **Accession*Conservation** | 137.744 | 14 | <0.001 |
| ***Z*** | **Accession** | 4.98 | 6 | 0.546 |
|  | **Conservation** | 7.151 | 2 | 0.007 |
|  | **Accession*Conservation** | 15.025 | 14 | 0.02 |
| **MC** | **Accession** | 438.199 | 6 | <0.001 |
|  | **Conservation** | 1845.743 | 2 | <0.001 |
|  | **Accession*Conservation** | 398.148 | 13 | <0.001 |
| **ROS** | **Accession** | 42.261 | 6 | <0.001 |
|  | **Conservation** | 246.989 | 2 | <0.001 |
|  | **Accession*Conservation** | 78.308 | 14 | <0.001 |

**Supplementary Table S5** Results of the generalized linear models (GLMs) performed on the levels of biochemical compounds MDA (malondialdehyde), tocopherols, free proline, and reducing sugars measured in the four selected accessions in their three conservation states. F, fresh seeds, harvested in 2019. A, seeds harvested in 2001 and kept in cold storage. R, seeds harvested in 2001 and conserved at room temperature. Y, yellow. G, green.

| **Parameter** | **Factors** | **Wald χ-squared** | **d.f.** | ***P* value** |
| --- | --- | --- | --- | --- |
| **MDA** | **Accession** | 177.029 | 3.000 | <0.001 |
|  | **Conservation** | 139.220 | 2.000 | <0.001 |
|  | **Accession*Conservation** | 54.472 | 6.000 | <0.001 |
| **Tocopherol** | **Accession** | 349.369 | 3.000 | <0.001 |
|  | **Conservation** | 69.546 | 2.000 | <0.001 |
|  | **Accession*Conservation** | 43.455 | 6.000 | <0.001 |
| **Proline** | **Accession** | 24.779 | 3.000 | <0.001 |
|  | **Conservation** | 46.787 | 2.000 | <0.001 |
|  | **Accession*Conservation** | 77.307 | 6.000 | <0.001 |
| **Reducing sugars** | **Accession** | 2097.451 | 3.000 | <0.001 |
|  | **Conservation** | 517.180 | 2.000 | <0.001 |
|  | **Accession*Conservation** | 272.290 | 6.000 | <0.001 |

**Supplementary Table S6** Results of differential scanning calorimetry (DSC) analysis: measures the temperature of glass transition (Tg) in the four selected accessions in their three conservation states. F, fresh seeds, harvested in 2019. A, seeds harvested in 2001 and kept in cold storage. R, seeds harvested in 2001 and conserved at room temperature. Y, yellow. G, green.

| **Accession** | **Conservation** | **Tg (°C)** |
| --- | --- | --- |
| Y1 | F | 72.88 ± 1.02 |
| Y1 | A | 78.78 ± 1.07 |
| Y1 | R | 69.33 ± 0.79 |
| Y2 | F | 77.25 ± 0.62 |
| Y2 | A | 75.96 ± 0.46 |
| Y2 | R | 80.30 ± 2.03 |
| G1 | F | 73.33 ± 0.59 |
| G1 | A | 79.10 ± 1.55 |
| G1 | R | 78.56 ± 1.23 |
| G4 | F | 75.40 ± 1.19 |
| G4 | A | 71.33 ± 1.42 |
| G4 | R | 76.02 ± 0.73 |

***
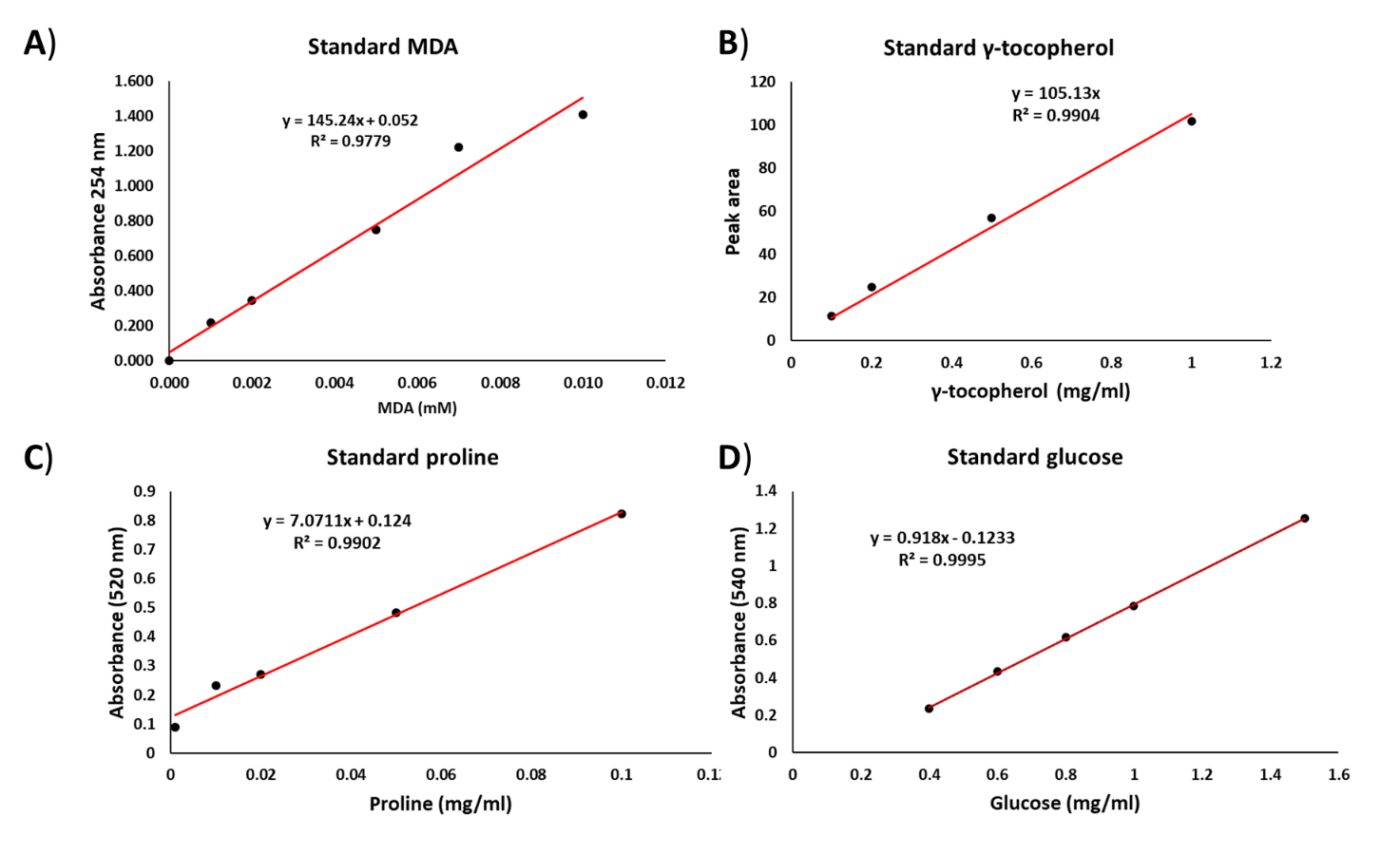
*Figures**

**Supplementary Fig. S1** **A)** Malondialdehyde (MDA) standard curve measured at 254 nm using an UV-visible spectrophotometer (UV-1800, Shimadzu, U.K.). **B)** The γ-tocopherol standard curve measured using the HPLC system (Kontron Instrument 420 system) equipped with a C18 column (250 x 4.6 mm, 5 μm). **C)** Proline standard curve measured at 520 nm using an UV-visible spectrophotometer (UV-1800, Shimadzu, U.K.). **D)** Glucose standard curve measured at 540 nm using an UV-visible spectrophotometer (UV-1800, Shimadzu, U.K.).

**
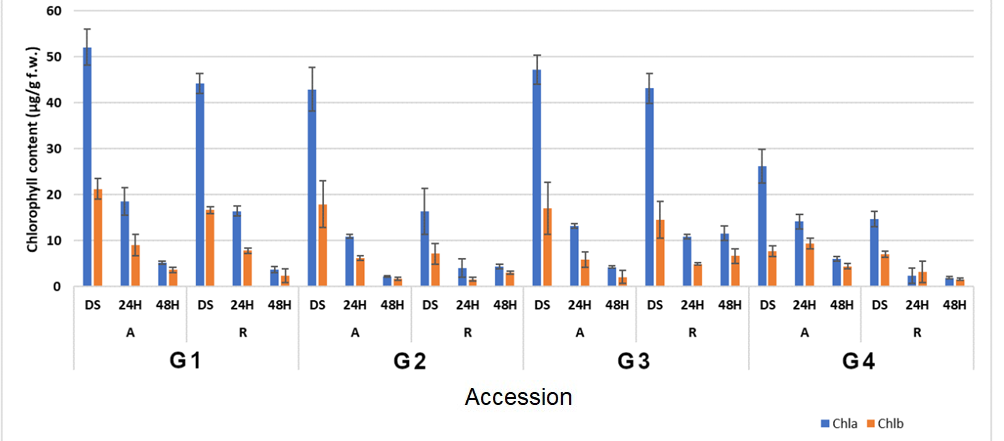
**

**Supplementary Fig. S2** Chlorophyll *a* (Chla) and chlorophyll *b* (Chlb) contents measured in the four green pea accessions G1, G2, G3, and G4 in both dry seeds (DS) and seeds imbibed for 24 h (24H) and 48 h (48H). A, seeds harvested in 2001 and kept in cold storage. R, seeds harvested in 2001 and conserved at room temperature conditions. f.w., fresh weight. Error bars represent the standard deviation of three biological replicates (10 seeds each).


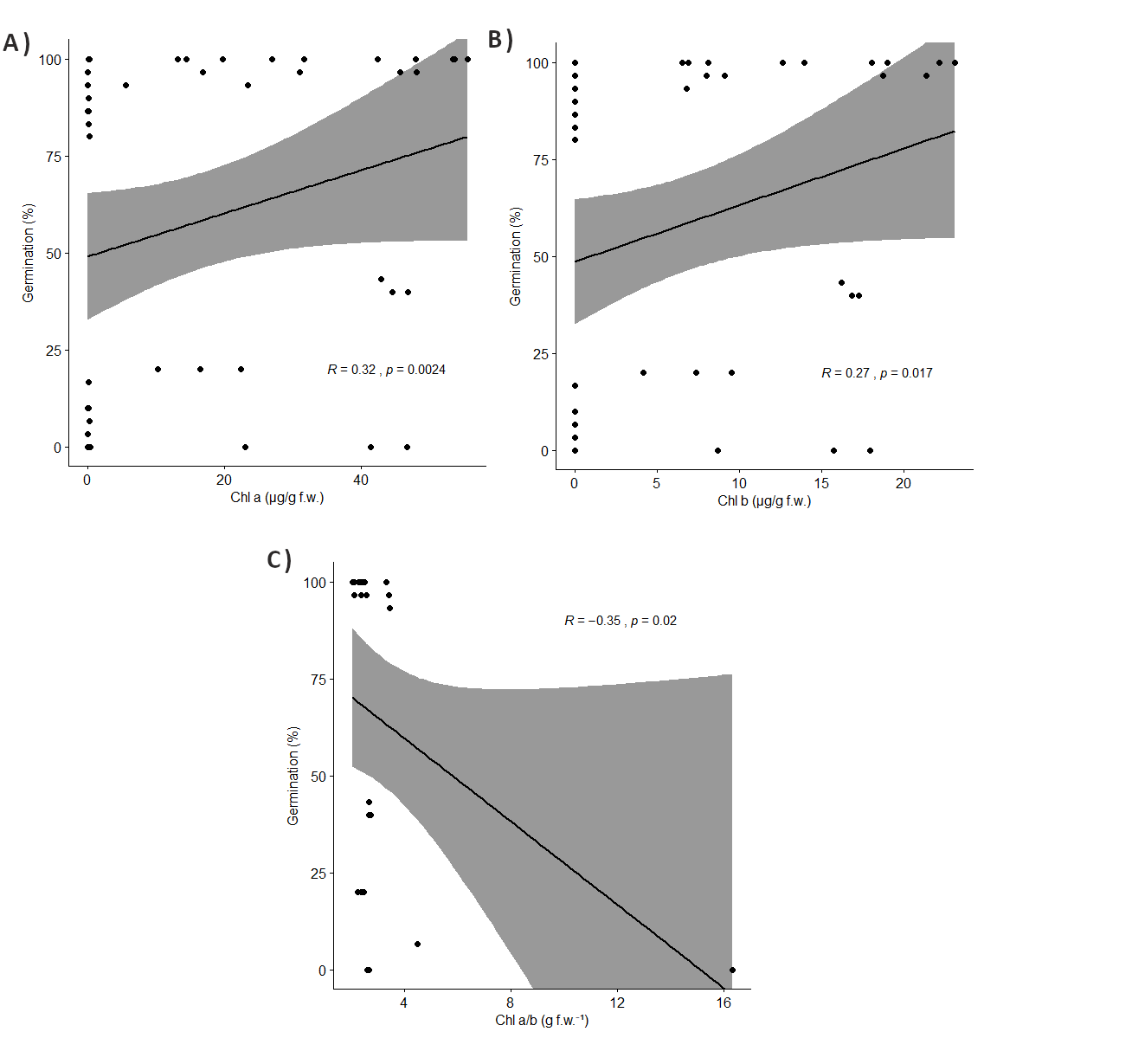


**Supplementary Fig. S3** Results of Kendall’s Tau-b test highlighting correlations between germination percentage and chlorophyll *a* (**A**), chlorophyll *b* (**B**), and chlorophyll *a*: chlorophyll *b* ratio (Chl a/b) (**C**). f.w., fresh weight.


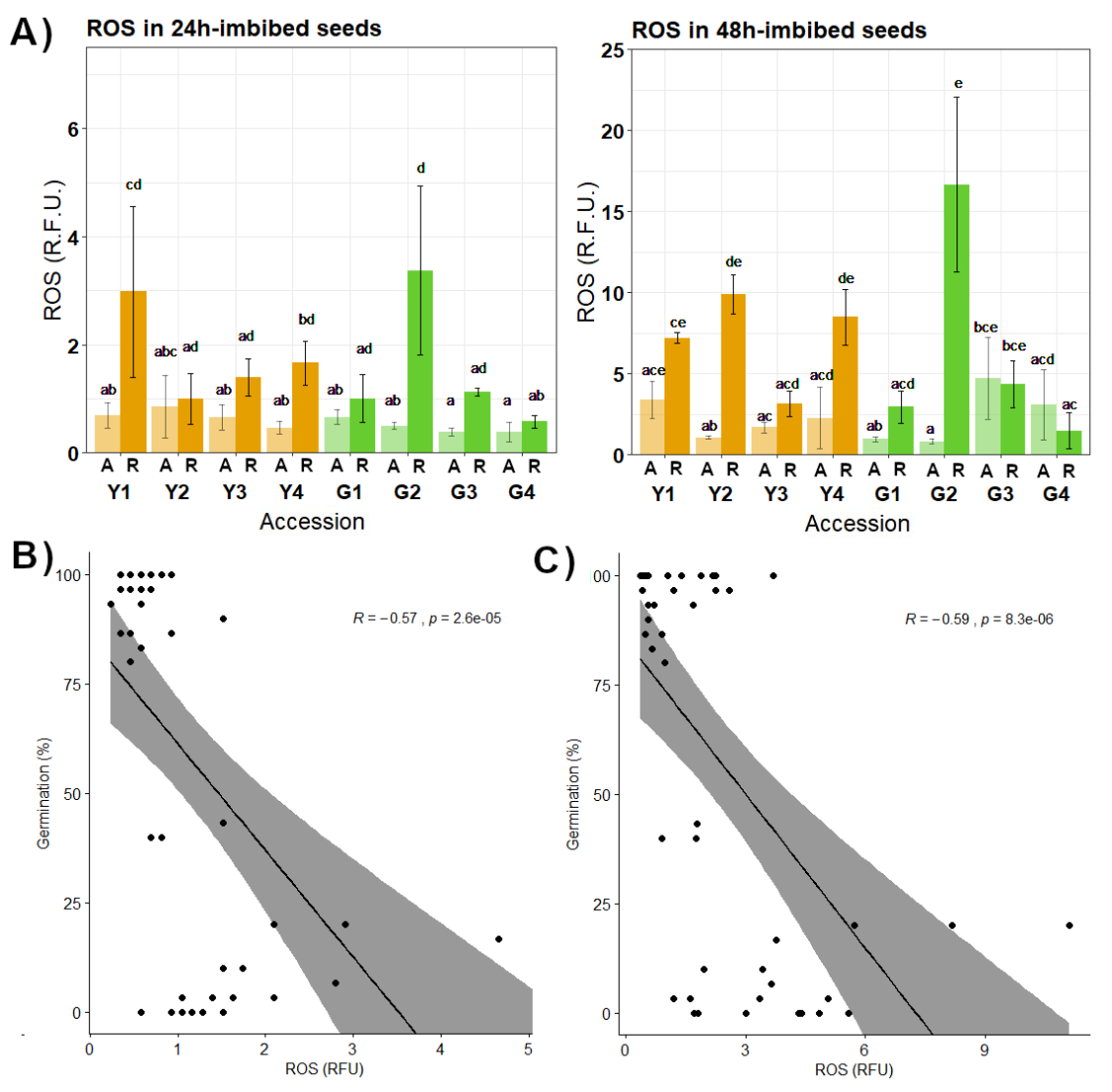


**Supplementary Fig. S4 A)** ROS levels measured in pea seeds of the yellow (Y1, Y2, Y3, Y4) and green (G1, G2, G3, G4) accessions, collected at 24 h and 48 h of imbibition, using the DCF-DA fluorescent dye. F, fresh seeds harvested in 2019. A, seeds harvested in 2001 and kept in cold storage. R, seeds harvested in 2001 and conserved at room temperature conditions. Letters above bars represent statistically significant differences (GLM with post-hoc Tukey’s test, *P* < 0.05) within the same variety. Error bars represent the standard deviation of three biological replicates (5 seeds each). R.F.U., relative fluorescence unit; ROS, reactive oxygen species; DCF-DA, dye 2’,7’-dichlorofluorescein diacetate. **B, C)** Kendall’s Tau-b correlation between ROS levels and germination profile of seeds from the yellow (Y1, Y2, Y3, Y4) and green (G1, G2, G3, G4) accessions, in imbibed seeds, (**B**) 24 h, and (**C**) 48 h.


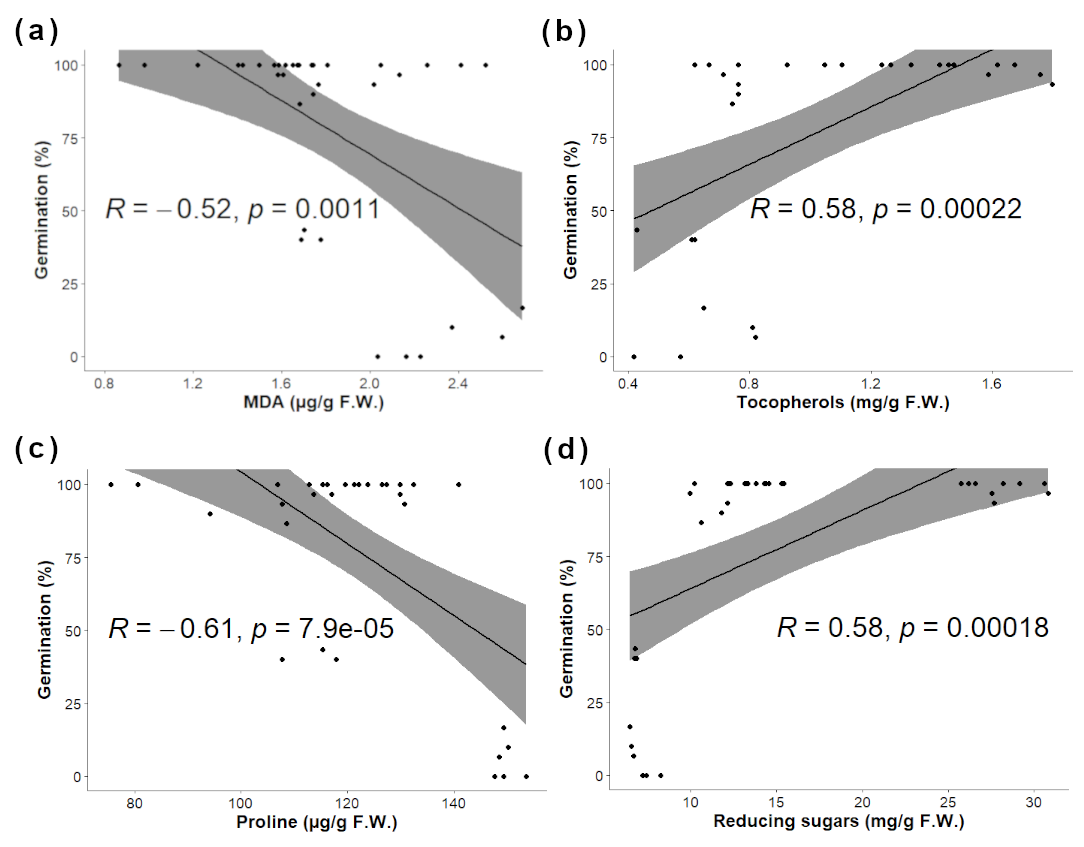


**Supplementary Fig. S5.** Kendall’s Tau-b correlations between germination percentage and (**A**) malondialdehyde (MDA), (**B**) tocopherols, (**C**) free proline, and (**D**) reducing sugars contents of seeds from the yellow (Y1, Y2) and green (G1, G4) accessions, in both fresh and aged (cold storage, room temperature conditions) seeds.


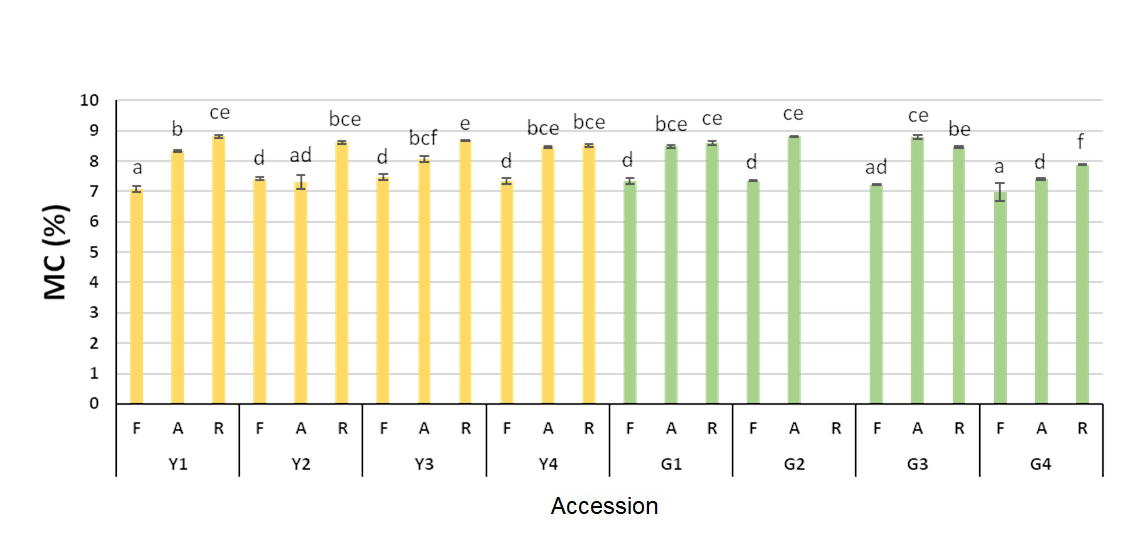


**Supplementary Fig. S6** Moisture content measured in pea seeds of the yellow (Y1, Y2, Y3, Y4) and green (G1, G2, G3, G4) accessions. F, fresh seeds harvested in 2019. A, seeds harvested in 2001 and kept in cold storage. R, seeds harvested in 2001 and conserved at room temperature conditions. Letters above bars represent statistically significant differences (GLM with post-hoc Tukey’s test, *P* < 0.05) within the same accession. MC, moisture content. Error bars represent the standard deviation of three biological replicates (30 seeds each).

*
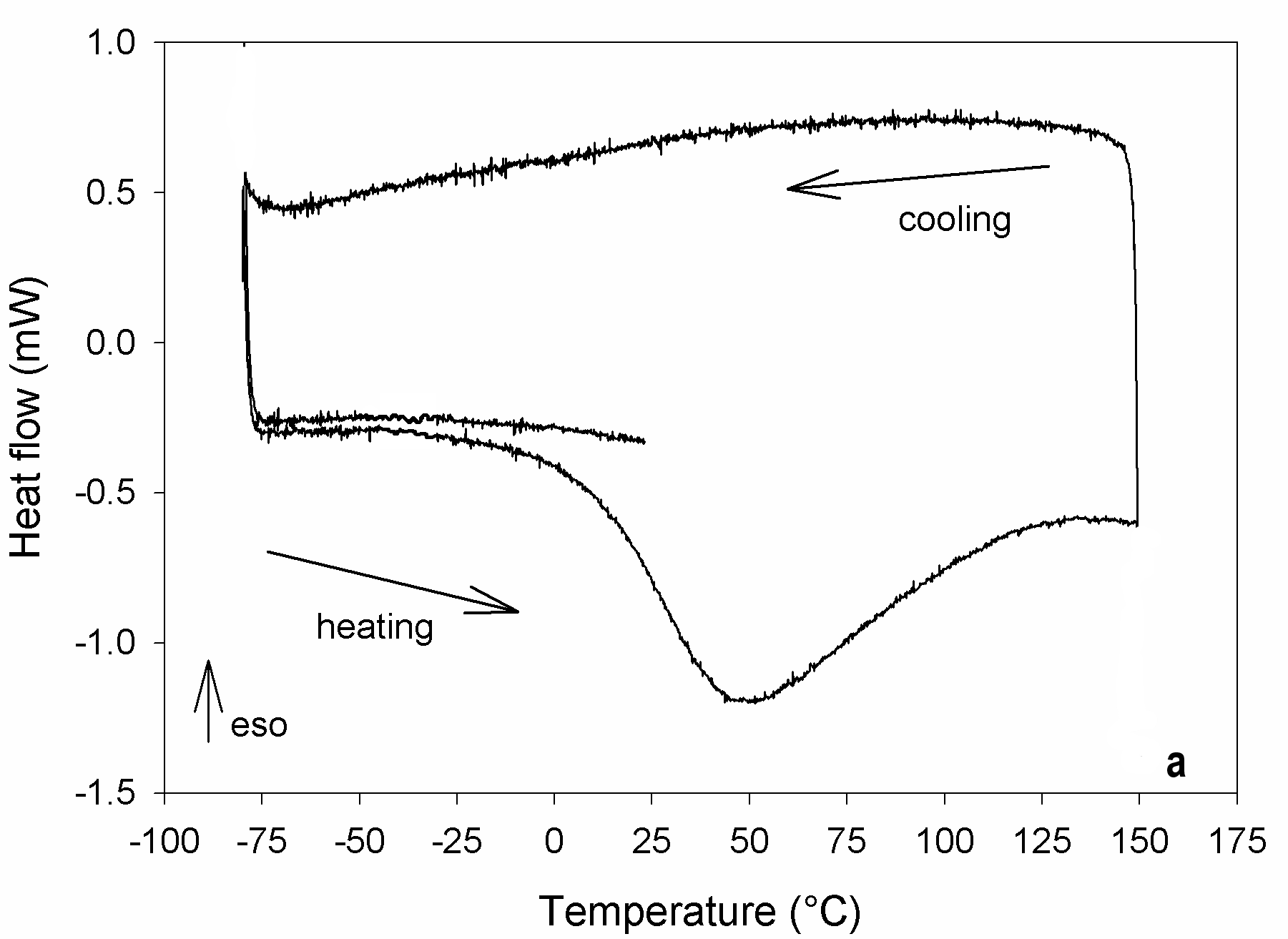
*

*
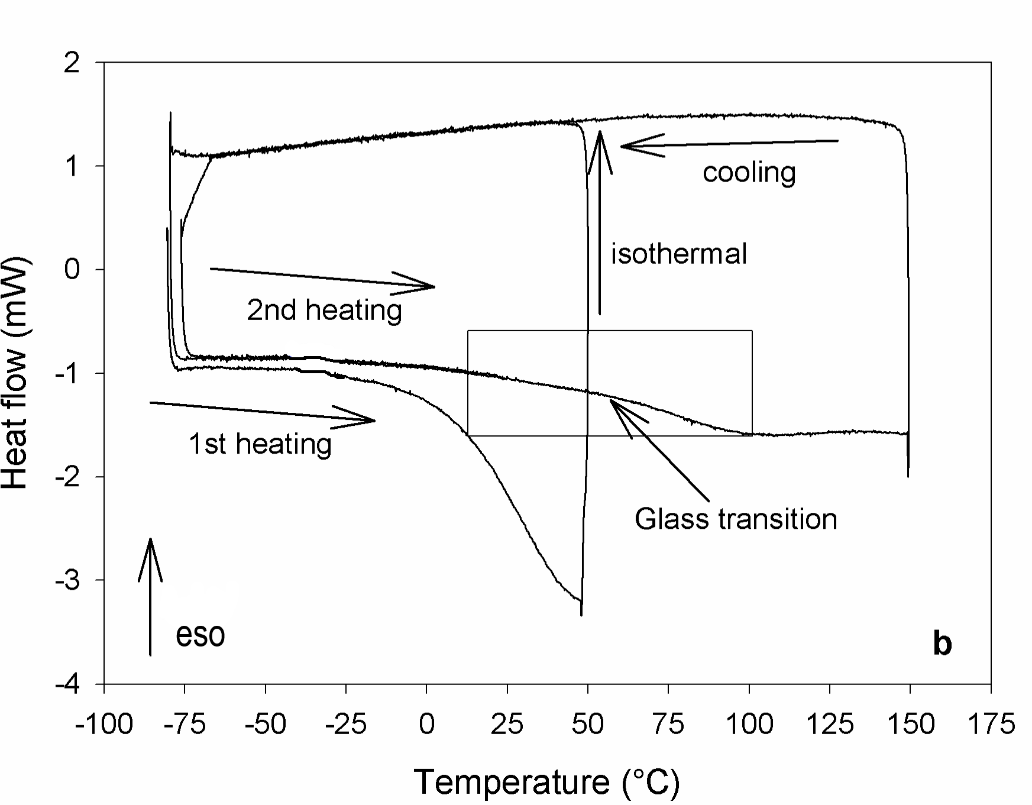
*

**Supplementary Fig. S7** Representative example of a calorimetric measurement for the A seeds (stored under controlled conditions) of the G1 accession obtained using a Q2000 Instrument (TA Instruments, New Castle, DE, USA). **(a)** heating and cooling cycle, highliting the endothermic peak due to moisture evaporation. **(b)** heating up to 50°C, isothermal stage for moisture evaporation, cooling and heating to catch the glass transition. The enlargement of figure b highlights the procedure followed by the software for the evaluation of the glass transition temperature: the operator points on the calorimetric curve the starting point and the ending point of the step, namely the beginning of the deviation from the base line and the ending point. The software evaluates the maximum slope of the step and draws a straight line tangent to the curve. The flex point on the calorimetric curve is the glass transition temperature.


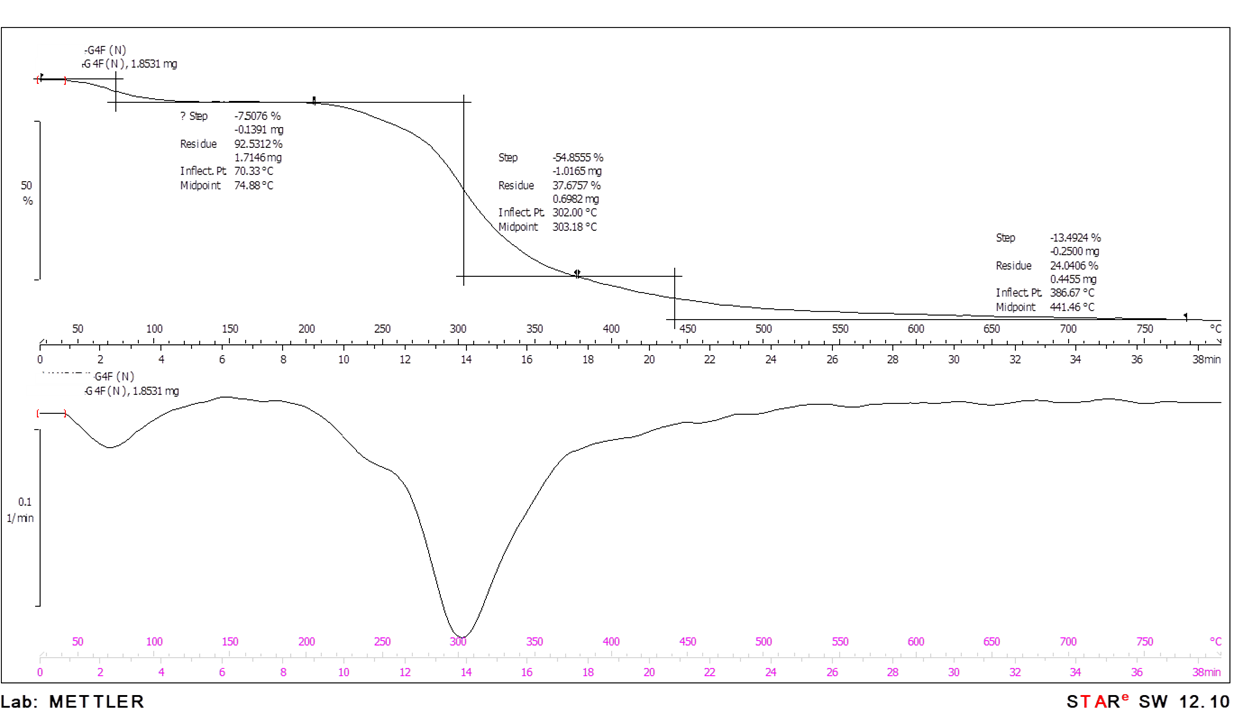


**Supplementary Fig. S8** Representative example of thermogravimetric measurements for the F seeds of the G4 accession obtained using a Mettler Toledo TGA 1 instrument under nitrogen.


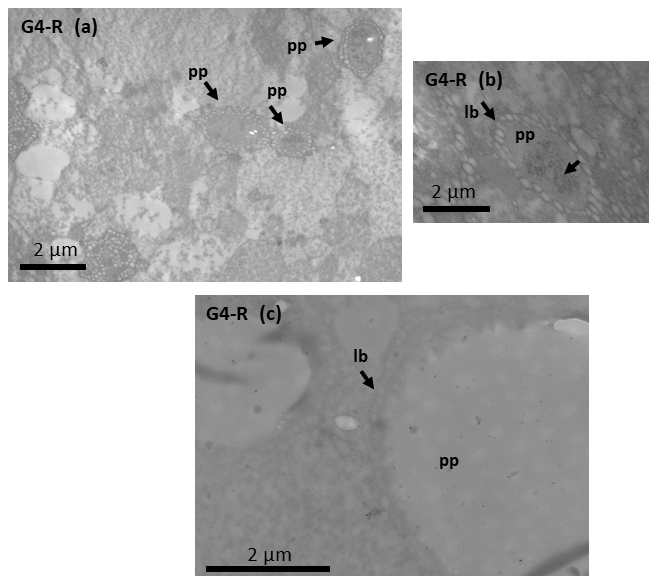


**Supplementary Fig. S9** Transmission electron microscopy analysis of sections of dry embryos excised from the G4 wrinkled seeds and subjected to periodic acid-Schiff (PAS) staining for 30 min in order to localize the occurrence of polysaccharides *versus* oligosaccharides in the pea embryo axes. **(a)** Cytoplasm region of a PAS-stained cell showing the distribution of some proplastids (pp, arrows). **(b)** Enlarged section of a PAS-stained cell showing a proplastid (pp) surrounded by several lipid bodies (lb) and the occurrence of weak dots inside the proplastid (arrow), resulting from the PAS mediated oxidation of polysaccharides. (c) Negative control (osmium ammine staining, without periodic acid treatment): enlarged section of a cell showing a proplastid (pp) and the surrounding lipid bodies (lb, arrow).


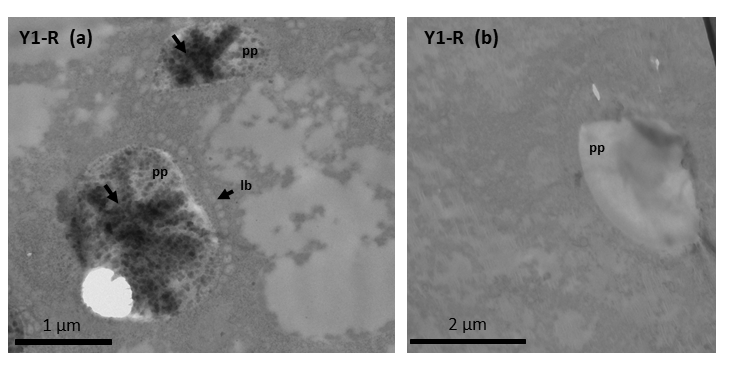


**Supplementary Fig. S10** Transmission electron microscopy analysis of sections of dry embryos excised from the Y1 seeds and subjected to periodic acid-Schiff (PAS) staining for 30 min in order to localize the occurrence of polysaccharides *versus* oligosaccharides in the pea embryo axes. **(a)** Cytoplasm region of a PAS-stained cell showing the distribution of some proplastids (pp), surrounded by several lipid bodies (lb) and the occurrence of intense precipitates inside the proplastid (arrow), resulting from the PAS mediated oxidation of polysaccharides. (**b**) Negative control (osmium ammine staining, without periodic acid treatment): enlarged section of a cell showing a proplastid (pp).
